# Supplementary material for: SODAR: managing multiomics study data and metadata
Source: Gigascience. 2023 Jul 27;12:giad052. doi: 10.1093/gigascience/giad052 (PMC10373112; doi:10.1093/gigascience/giad052)
Supplement: giad052_GIGA-D-22-00194_Original_Submission [file giad052_giga-d-22-00194_original_submission.pdf]

GigaScience

SODAR: enabling, modeling, and managing multi-omics integration studies

--Manuscript Draft--

**Manuscript Number:**	GIGA-D-22-00194
**Full Title:**	SODAR: enabling, modeling, and managing multi-omics integration studies
**Article Type:**	Technical Note
**Funding Information:**	
**Abstract:**	Scientists employing omics in life science studies face challenges such as the modeling of multi assay studies, recording of all relevant parameters, and managing many samples with their metadata. Further, they must manage many large files that are the results of the assays or subsequent computation. Scientists from diverse backgrounds also have different demands for interfacing with the data, ranging from computational users that need programmatic or command line access whereas non-computational users need graphical interfaces. We introduce SODAR, the system for omics data access and retrieval. SODAR is a software package that addresses the points above by providing a web-based graphical user interface for managing multi assay studies and describing them using the open ISA (Investigation Study Assay) data model and ISA-Tab file format. Data storage is handled using the iRODS data management system which effortlessly handles many files and large amounts of data. SODAR also provides programmable APIs and command line access for metadata and to file storage. SODAR supports multi-omics integration studies and can be easily installed. The software is written in Python 3 and freely available at https://github.com/bihealth/sodar-serverunder a permissive open-source license.
**Corresponding Author:**	Mikko Nieminen, M.Sc.<br>Berlin Institute of Health at Charite<br>Berlin, Berlin GERMANY
**Corresponding Author Secondary Information:**	
**Corresponding Author's Institution:**	Berlin Institute of Health at Charite
**Corresponding Author's Secondary Institution:**	
**First Author:**	Mikko Nieminen, M.Sc.
**First Author Secondary Information:**	
**Order of Authors:**	Mikko Nieminen, M.Sc.
	Oliver Stolpe
	Mathias Kuhring
	January Weiner
	Patrick Pett
	Manuel Holtgrewe
	Dieter Beule
**Order of Authors Secondary Information:**	
**Additional Information:**	
**Question**	**Response**
Are you submitting this manuscript to a special series or article collection?	No
**Experimental design and statistics**	Yes

Powered by Editorial Manager® and ProduXion Manager® from Aries Systems Corporation

Full details of the experimental design and statistical methods used should be given in the Methods section, as detailed in our Minimum Standards Reporting Checklist. Information essential to interpreting the data presented should be made available in the figure legends.<br><br>Have you included all the information requested in your manuscript?	
**Resources**<br><br>A description of all resources used, including antibodies, cell lines, animals and software tools, with enough information to allow them to be uniquely identified, should be included in the Methods section. Authors are strongly encouraged to cite Research Resource Identifiers (RRIDs) for antibodies, model organisms and tools, where possible.<br><br>Have you included the information requested as detailed in our Minimum Standards Reporting Checklist?	Yes
**Availability of data and materials**<br><br>All datasets and code on which the conclusions of the paper rely must be either included in your submission or deposited in publicly available repositories (where available and ethically appropriate), referencing such data using a unique identifier in the references and in the "Availability of Data and Materials" section of your manuscript.<br><br>Have you have met the above requirement as detailed in our Minimum Standards Reporting Checklist?	Yes

Powered by Editorial Manager® and ProduXion Manager® from Aries Systems Corporation

Manuscript

Click here to access/download;Manuscript;2022_Nieminen_SODAR_Manuscri

SODAR: enabling, modeling, and managing multi-omics integration studies

Mikko Nieminen[1], Oliver Stolpe[1], Mathias Kuhring [1], January Weiner 3rd [1], Patrick Pett[1], Dieter Beule[*,1], and Manuel Holtgrewe [*, 1]

[1]Berlin Institute of Health at Charité – Universitätsmedizin Berlin, Core Unit Bioinformatics (CUBI),

Charitéplatz 1, 10117 Berlin, Germany

[*]These authors contributed equally

Abstract

Scientists employing omics in life science studies face challenges such as the modeling of multi assay studies, recording of all relevant parameters, and managing many samples with their metadata. Further, they must manage many large files that are the results of the assays or subsequent computation. Scientists from diverse backgrounds also have different demands for interfacing with the data, ranging from computational users that need programmatic or command line access whereas non-computational users need graphical interfaces.

We introduce SODAR, the system for omics data access and retrieval. SODAR is a software package that addresses the points above by providing a web-based graphical user interface for managing multi assay studies and describing them using the open ISA (Investigation Study Assay) data model and ISA-Tab file format. Data storage is handled using the iRODS data management system which effortlessly handles many files and large amounts of data. SODAR also provides programmable APIs and command line access for metadata and to file storage.

SODAR supports multi-omics integration studies and can be easily installed. The software is written in Python 3 and freely available at https://github.com/bihealth/sodar-server under a permissive open-source license.

Keywords: Scientific Data Management, ISA-Tab, iRODS

1. Introduction

Modern studies in the life sciences often involve running multiple “omics” assays (*e.g.*, genomics, proteomics, and metabolomics). Such studies require careful planning, data collection, data analysis and integration of data. An example of such complex study is (Esterhuyse *et al.,* 2015) in infection biology, which will be used as an example below. Ideally, scientists are supported by a detailed modeling of each of the involved steps to keep track of the status of data acquisition as well as relevant factors and confounders.

The most comprehensive standard for describing study metadata is the ISA-Tab format (Sansone *et al.*, 2012) which allows modeling studies with multiple samples and assays. ISA-Tab is a tabular file format that allows users to model each processing step with each intermediate result and annotate each of these with arbitrary metadata. Alternatives include Portable Encapsulated Projects (PEP) by Sheffield *et al.,* (2021).

Another characteristic of modern omics studies is that they generate large volumes of data ranging from few gigabytes for mass spectrometry to tens of gigabytes for genomics sequencing to terabytes for imaging/microscopy. Such data sets must be managed both in the intrinsic complexity of their structure and metadata, generated raw data, and subsequent computational processing results. In the simplest case, data can just be stored using file systems or object storage systems. More advanced systems such as Shock (Bischof *et al.*, 2015) or dCache (Ernst *et al.*, 2001) also allow for storing metadata and distributing the data to a “data grid” over multiple servers. iRODS (Hedges *et al.*, 2009; Chiang *et al.*, 2011) adds even further features such as running programs within the data system and providing integration with arbitrary authentication systems.

Once published, multi omics study data is often deposited in public data portals such as BioSamples database (Courtot *et al.*, 2022). However, before completion and publication researchers need to capture experiment and sample metadata as well as the generated mass data in private systems. Systems for capturing data and experiment metadata include ELN (laboratory notebooks, cf. Higgins *et al.*, 2022). Raw experiment data is commonly stored in LIMS (laboratory information management systems). Few published dedicated systems for storing both omics mass and metadata are available such as qPortal (Mohr *et al.*, 2018; Cuellar *et al.*, 2022) which is itself based on OpenBIS (Bauch *et al.*, 2011).

In this manuscript, we introduce SODAR (the system for omics data access and retrieval). SODAR combines the modeling of studies and assays using the ISA-Tab standard with handling of mass data storage using iRODS (integrated rules-oriented data system). We demonstrate the features of SODAR with a multi-omics use case. More example projects are available in the SODAR online demo at https://sodar-demo.cubi.bihealth.org.

2. Results

We first describe the SODAR system. We then perform a qualitative comparison of SODAR with similar software. We then describe the overall process of using SODAR, provide a multi-omics use case and show realizations of the abstract steps described above.

2.1 Resulting System Overview

Figure 1 shows the components of the SODAR system. SODAR Server contains the main system logic, providing a web-based user interface (UI) and REST APIs for managing metadata. Mass data storage is implemented using iRODS, and the common WebDAV protocol is provided by Davrods. Non-

computational users can interface with SODAR using the graphical UI, whereas computational users can use command line interfaces and REST APIs from scripts and other external software.

Figure 1 SODAR system with its components and actors. The figure illustrates how actors interact with SODAR and iRODS through different APIs.

2.1 Comparison of Features

Our motivation for the development of SODAR was the unavailability of an appropriate system for both serving for modeling experiments and storing meta and mass data. We collected systems with similar features in Table 1 and compared them to SODAR using categories for key SODAR features.

Table 1 Major features differentiating SODAR from other systems. See main text for a detailed description.

	SODAR	LIMS	ELN	Study Databases	File Systems	MOLGENIS	qPortal
**Donor Annotation**	✓	(✓)	▪	✓	▪	✓	✓
**Sample Annotation**	✓	(✓)	▪	✓	▪	✓	✓
**Process Annotation**	✓	✓	▪	×	▪	✓	✓
**Ontology Support**	✓	×	(×)	(✓)	×	✓	▪
**Arbitrary Experiments**	✓	(✓)	▪	×	▪	✓	✓
**Multi-Omics Support**	✓	×	▪	×	▪	✓	✓
**Large File Support**	✓	×	×	×	▪	×	✓
**Custom Installation**	✓	✓	✓	×	✓	✓	✓
**REST API**	✓	(✓)	(✓)	(✓)	×	✓	×

Code: structured (✓), unstructured (▪), missing (×); parentheses: depending on implementation.

The table compares SODAR to laboratory information management systems (LIMS), electronic laboratory notebooks (ELN), study databases such as the one provided by NCBI dbGAP or sequence read archive, using (network) file systems for file storage, and the MOLGENIS toolkit. The table shows

whether certain attributes can be captured with structured means (e.g., enforcing data structures or controlled vocabulary) or unstructured means (plain text).

The following features are important for a comprehensive system for Omics data management:

Donor (Sample Source / Patient) Annotation Annotating probands with information such as population background, disease type or cancer stage is important for capturing elementary features and confounding factors.

Sample/Process Annotation Samples (and analytes) as well as processes should be annotated according to information to capture relevant parameters and measures.

Ontology Support A key feature for capturing information in a structured way is the support of ontologies.

Arbitrary Experiments It is important whether systems allow the modeling of arbitrary experiments or are limited to certain kinds (e.g., LIMS often are flexible in principle but are usually configured for streamlined web-lab processes).

Multi-Omics Experiments Relatedly, SODAR allows for capturing that the same set of samples has been subjected to different experiments or assays (“multi-omics support”).

Large file support is another key feature and enables storing mass data for the experiment results.

Custom Installation Finally, we captured whether a custom installation using on-premises hardware is possible with the different systems.

REST APIs in data management systems enable many powerful applications such as integration with existing database systems or integration into third-party client software.

All these features are supported by SODAR, making it a unique framework for supporting users in multi-omics data management. Importantly, SODAR provides REST APIs and hyperlinks into its metadata and

mass data repositories and thus can be easily integrated with other systems. Note that each of the systems considered caters to a different niche and SODAR is not meant to replace any of the other software packages or outclass them in their niche.

2.2 General Sodar Process

The general workflow in using SODAR for managing data and metadata is shown in Figure 2. We distinguish between the roles “data steward” and “experimentalist”, however in some cases one person might have both roles. The former are responsible for creating the overall structure of the data, while the latter are responsible for entering the actual data into the system.

Data stewards are users who are experienced with using ISA-tab files and in our use case are bioinfsormaticians working in the core unit. They are responsible for modeling the experiments in the ISA-tab format as “sample sheets” with the overall experimental design. They generally also maintain a library of sample sheet templates for common use cases. With experienced experimentalists the steward might just create the general structure of the experiment. When necessary, the steward might pre-create the sample sheet with a full skeleton of all planned samples and processes and IDs together with the experimentalists.

Experimentalists are users who are more concerned with completing the data in the sample sheet rather than in the creation of its structure. When the full sample sheet is created together with the stewards, they might only verify the structure with the information of their experiments (that is stored in a ELN, for example) and fill in some measurements in sample sheet cells (e.g., concentration measurements). More trained and experienced experimentalists will also create new rows for samples.

Of course, using the REST API of SODAR it is possible to automate all these tasks. For example, an integration with a LIMS system could automatically create samples as they are processed in the wet-lab while measurements could be written to SODAR from the LIMS or from an integration of an ELN system. SODAR provides a set of templates for common experiment types, but users can also use external software such as ISA-tools (Sansone *et al.*, 2012).

Figure 2 SODAR (meta) data management workflow. The workflow scheme is divided into steps attributed to a data steward (blue) who manages the overall data schema and experimental user (green) who enters the actual data or uploads files.

In general, there are two types of data in the SODAR system. The metadata, which includes information about the samples, procedures, analysis, experimental scheme etc., is stored in the ISA-tab and can be edited within the SODAR system using a GUI or manipulated with the REST API. In contrast, the experimental data – results of the measurements, for example FASTQ files for sequencing or mass spectrometry XML files – can be uploaded to SODAR using a separate two-stage GUI or uploaded using the REST API.

For each experiment, SODAR manages a corresponding directory in the iRODS data repository. Each sample/analyte is associated with a sub directory in this repository and users can upload data for individual samples/analytes or whole experiments. To enforce checking the integrity and correctness of the data, users do not manipulate data directly in the data repository. Instead, they first upload data to “landing zones” where they have full read-write access. SODAR enforces certain best practices such as requiring a checksum for each uploaded file. Once the uploading user is content with the uploaded data, they submit the landing zone for import into the project data repository. SODAR checks the uploaded

data and moves data to the project data repository where it is immutable for the general users. Users can submit requests for deleting data which has to be confirmed by a project owner or delegate.

As mentioned above, SODAR associates the data in the iRODS project data repository with the samples and materials based on the directory names in iRODS. Users can easily access any file in the projects that they have access to via the SODAR UI, WebDAV which allows mounting the storage on their desktop machine, or the iRODS protocol and command line tools. The metadata can be exported to ISA-Tab files. Uploading ISA-Tab files is also allowed, if the user has existing files or wants to use external applications for editing them. We further added special support for the IGV genome browser (Robinson *et al.,* 2011) into SODAR. For germline and cancer DNA sequencing experiments, SODAR can generate IGV session files that can be directly opened from the browser. Users can thus open IGV with the variant and NGS read alignment files for whole families (germline) or tumor-normal pairs (cancer) with a single click.

To conclude, SODAR supports computational and experimental users with functionality to model their experiments, upload resulting files, and accessing the files through effective and easy-to-use means.

2.3 Use Case Description

In the following sections, we will show how SODAR can be used for supporting multi-omics studies using the one by Esterhuyse *et al. (*2015) as an example. To be clear, this study was originally not performed using SODAR. We will illustrate the modeling, data import, and upload steps.

For their research, Esterhuyse *et al.* (2015) were looking at methylation, transcriptomics, and proteomics to identify tuberculosis (TB) biomarkers of latently infected subjects and TB patients. They recruited n=8 subjects with latent *M. tuberculosis* infection and the same number of TB patients. Blood was collected from each patient and then subjected to DNA methylation and transcription analysis using

microarrays and proteomics analysis was performed using mass spectrometry. The resulting data was then subjected to statistical analysis and led to the published article.

2.3.1 Modeling

When applying SODAR, our group of bioinformaticians and biostatisticians meets with the experimentalists and discuss sample sizes and suitable assay types. To simplify the description we describe the people with the computational/statistical knowledge as having the “biostatistician” role. In the case of the given study, we would decide together with the experimentalists on the given sample types given availability of funding and donors. Our work focuses on commonly used assays which focus on NGS-based ones but also include certain proteomics and metabolomics assays used by labs that we are collaborating with regularly.

In the case of the TB study, a member of our group would take the role of the data steward and we would first create the sample sheet structure of the blood sampling itself. In ISA terminology, the blood donors are “source” while the collected blood is “samples”. The data steward would define relevant source factors (e.g., acute or latent infection) and important confounders (such as age) together with the biostatistician and the experimentalists. The biostatisticians are generally trained as data stewards and thus have both roles but they might also talk to other data stewards in case of questions.

We would then continue to model the relevant parts of the experiment. Important modeled experiment steps may be the extraction of analytes such as RNA, the measurements themselves, and the vendor software and version used for the primary data analysis. Important properties of the occurring analytes (“materials” in ISA terminology) and processes include RNA concentrations, the used microarrays including lot numbers, as well as software versions.

2.3.2 Sample Metadata Definition/Data Entry

Data stewards use ontologies and controlled vocabularies where possible. This would be discussed with the experimentalists and suitable terms would be agreed upon together. In the case of ambiguity, it makes sense to attempt to use the same term for the same real-world object across projects as to improve data reusability.

In the work of our group, we would generally fill one or two example rows with our collaborators but then hand it over to them to fill in the actual metadata. The resulting sample sheet would then be iteratively improved through review by the data stewards and further discussion with the experimentalists. Adjustments may include adding further columns (e.g., for measurements) to the sample sheet, adjustment of the used ontology terms, and adding or removing sample rows.

Of course, projects generally aim at having a stable plan of the work but adjustments may be required over time. Common reasons for such adjustments are drop-outs during certain assays, additional measurements becoming required, or having to perform additional assays or use additional sample during review for publication. SODAR allows such adjustments to the data it handles over time and stores the sample sheet versions after each change. It also includes a tool for comparing sample sheets to inspect the performed changes.

2.3.3 Raw Data Import & Raw Data Access for Processing & Result Import after Processing

Eventually, the experimentalists perform the modeled wet-lab steps, measurements, and primary data analysis. The resulting raw data is imported into SODAR by first creating a landing zone, uploading the data, either done by lab technicians or us bioinformaticians, and then moving the data of the editable landing zone to the read-only per-project data repository.

The biostatisticians obtain the metadata and mass data created by the experimentalists and download them using the web interface or command line interfaces/REST APIs. The data is analyzed as appropriate. Resulting data and reports are then uploaded to SODAR, again using landing zones.

This is usually followed by a series of discussions with the experimental partner where the analysis is refined and the subsequently generated resulting data files and reports are deposited in the read-only SODAR per-project data repository. Of course, such iterations might include having to update the sample sheets as described in Section 2.2.2 and subsequent reanalysis. In the case that files in the read-only data repository are to be replaced, they have to be removed before which can be done using a two-step/four-eyes process where users can create a deletion request that the project owner has to confirm.

2.3.4 Resulting Data Access

The read-only project data repository is intended for long-time storage. All data is available to the experimentalist in a self-service fashion such that they can re-use all data in subsequent studies or access the intermediate and final results to answer requests for data sharing or questions regarding their publication.

2.4 Internal Usage Statistics

In the spirit of "eating your own dog food", we have been using SODAR in our group's projects for the past four years. Table 2 gives summary statistics of data and metadata stored in our internal instance as well as the diversity of projects. We thus tested SODAR extensively in a real-world setting and use it daily as our main storage for all our project data and metadata.

Table 2 Summary statistics of project type and count, sample count, and user count as well as mass data file count and total size in our internal instance of SODAR.

**Projects**	313
**Users**	324
**Samples**	18 690
**Total File Count**	187 629
**Total File Size**	357 TB

Statistics collected in July 2022

3. Methods

SODAR is implemented in Python 3 using the Django web framework and Django REST Framework. Reusable components have been extracted into the library SODAR Core (Nieminen *et al.*, 2020). ISA-Tab format manipulation has been implemented using AltamISA (Kuhring et al., 2019).

3.1 Project Organisation, Authorization Structure, and LDAP Integration

SODAR uses the concept of “projects” for organizing all data. Projects have a unique identifier and some basic metadata such as title, description, etc. Projects can be organized in a tree structure using the concept of “categories” that can contain projects or other categories. Each project has a single owner, who can assign themselves a delegate for managing the project. Further users can be granted access to the project either in a read-write (contributor) or a read-only fashion (guest) using role-based access control.

SODAR can be configured to be run standalone or integrated with LDAP servers (including Microsoft ActiveDirectory) for providing authentication information, where authentication refers to checking the identity of a user based on their username and password.

3.2 iRODS integration

SODAR automatically manages user access to projects in iRODS. This is done by creating an iRODS directory and user group for each project. The group is given access to the directory and group membership is synchronized between the SODAR database and iRODS.

Further, SODAR creates a sub directory for each study and assay from the ISA model of the project. Users can use the landing zone mechanism for adding files for each sample/analyte or add them for the whole study or assay. Users can add thus add data for an arbitrary number of assays for each sample and original donor or specimen.

The files can be accessed either directly through the iRODS protocol or using the WebDAV protocol through the Davrods (Smeele&Smeele, 2016) software. The latter allows users to access the storage as a network drive on their desktop computers. Since WebDAV is HTTP based, users can also make data available to genome browsers such as IGV or UCSC Genome Browser. Moreover, it is generally easy to access data through an organization's firewall and proxies without intervention of IT departments.

Optionally, SODAR allows the management of iRODS "tickets", which allow for access based on randomly generated tokens instead of user login. This way, users can upload genome browser tracks to SODAR/iRODS and create public URL strings to access them and share them with users that do not have access to the full project (or do not even have an account in SODAR).

3.3 Sample Sheet Editor, Import, Export

Sample sheets can be included into SODAR projects by either importing existing ISA-Tab files or template-based creation. A single project corresponds to an "investigation" in the ISA-Tab naming convention. When importing, the user can upload a Zip archive or a set of individual ISA-Tab files. For creating sample sheets from templates, the user needs to fill in certain details in the SODAR UI. SODAR

provides multiple built-in templates for, e.g., generic RNA sequencing, germline DNA sequencing and mass spectrometry-based metabolomics. After import or creation, the sample sheets are stored in an object-based format in the SODAR database for easy search and modification. In the UI, they are presented to the user as spreadsheet-style study and assay tables.

The user can edit sample sheets in the SODAR UI (Sup. Figure 3). Cells in the study and assay tables can be edited in a similar fashion to a spreadsheet application. For each column, the project owner or delegate can define the accepted format, value choices, value ranges, regular expressions for accepted values, and other settings depending on the column type. This ensures the validity of data and its compatibility with the study's requirements and conventions.

SODAR supports ontology term lookup for cell editing. Commonly used ontologies such as HPO, OMIM and NCBITaxon can be uploaded into SODAR in the OBO and OWL formats for local querying, without the need to rely on third party APIs. Manual entering of ontology terms is also allowed. It is possible to include multiple ontology terms in a single cell and one or several ontologies can be used in a single column.

In addition to cell editing, the user can insert and remove rows for study and assay tables. Cells for existing sources, samples, materials or processes are auto filled by the editor when including a new row. Similarly, if multiple rows contain references to the same entity, all related cells are automatically updated in the tables when modifying them on a single row. SODAR validates all edits using the AltamISA parser (Kuhring et al. 2019). This ensures the validity and ISA-Tab compatibility of the sample sheets at all times.

When editing sample sheets, old sheet versions are stored as backup. These versions can be compared and restored in case of mistakes, as well as exported from the system. SODAR allows for sample sheet

export in the full ISA-Tab TSV format, or simplified Excel tables. Replacing existing sheets with versions modified outside of SODAR is also supported.

3.4 Integrating SODAR Core based sites

Several subcomponents of the SODAR server such as project and user management have proven to be useful in other contexts. We have extracted them into the SODAR Core library (Nieminen *et al.*, 2020) which forms the foundation of other projects such as VarFish (Holtgrewe *et al.*, 2020) and Kiosc (Stolpe *et al., Submitted*). Using a common library for projects and access management has several advantages and enables the integration of VarFish and Kiosc with SODAR.

SODAR can be configured to work as a “source” site. Applications based on SODAR Core can then be configured as “target” sites of the source site. Projects and access to users will then be synchronized to target sites. This allows us to manage sample and experiment definitions in SODAR and uploading corresponding variant data to VarFish. VarFish can then use the REST APIs defined by SODAR for synchronizing sample metadata (such as phenotype terms) directly from SODAR. Similarly, users can upload mass data files into the iRODS data repository and create access tokens to them in SODAR. These tokens can be used to provide data visualization applications in Kiosc with data access via HTTP and iRODS protocols or even external applications such as UCSC genome viewer (Navarro Gonzalez *et al.* 2021).

3.5 SODAR Administration

We provide a straightforward way to install SODAR and related components (SODAR, iRODS, Davrods, and supporting database servers) and maintain such an installation based on Docker containers and

Docker compose. Detailed installation instructions can be found in the sodar-docker-compose repository linked to in the section “4.2 Source Code Availability”.

The whole system can be set up using an external LDAP/ActiveDirectory server for users and credentials or as an alternative in a standalone fashion where SODAR hosts this information. Further, users can of course use their external iRODS installation. Finally, SODAR features administrator dashboards for providing statistics about projects and usage of storage resources.

4. Data and Source Code Availability

4.1 Data Availability

A demonstration instance with all data is available at https://sodar-demo.cubi.bihealth.org. All source code is available from https://github.com/bihealth/sodar-server. All metadata is available in ISA-tab format from https://github.com/bihealth/sodar-paper.

4.2 Source Code Availability

Project name: sodar-server

Project home page: https://github.com/bihealth/sodar-server

Operating system: Linux/Unix

Programming language: Python

License: MIT

RRID: SCR_022175

Biotools: biotools:sodar

5. Supplemental Figures

Figure_3_Supplementary_Material.pdf: SODAR sample sheet editor screenshots with major features annotated.

6. Abbreviations

API:	Application Programmable Interface
CUBI:	Core Unit Bioinformatics
ELN:	Electronic Laboratory Notebook
GUI:	Graphical User Interface
HPO:	Human Phenotype Ontology
HTTP:	Hyper Text Transfer Protocol
IGV:	Integrated Genomics Viewer
IRODS:	Integrated Rules-Oriented Data System
ISA:	Investigation Study Assay
LDAP:	Lightweight Directory Access Protocol
LIMS:	Laboratory Information Management System
MIT:	Massachusetts Institute of Technology (also commonly used with "MIT license")
NCBI:	National Center for Biotechnology Information
OBO:	Open Biological and Biomedical Ontologies
OpenBIS:	Open Biology Information System
OWL:	Web Ontology Language
PAM:	Pluggable Authentication Mechanism
PEP:	Portable Encapsulated Projects
SODAR:	System for Omics Access and Retrieval
TB:	Tuberculosis
TSV:	Tabular Separated Values
UCSC:	University of California Santa Cruz
WebDAV:	Web-based Distributed Authoring and Versioning)

7. Competing Interests

The authors declare that they have no competing interests.

7.1 Funding

The authors have no funding to declare beyond their organization.

7.2 Author's Contributions

Conceptualization: MN, MH, DB. Funding Acquisition: DB. Methodology: MH, MN. Project Administration: MH, DB. Resources: DB. Software: MN, MH, OS, PP. Supervision: MH, DB. Writing and Editing: all authors

8. Acknowledgements

The authors thank all internal users, in particular CUBI members for their feedback. Some icons from OpenMoji.org were used in the figures.

9. References

1. Brademan DR, Miller IJ, Kwiecien NW, Pagliarini DJ, Westphall MS, Coon JJ, et al.. Argonaut: A Web Platform for Collaborative Multi-omic Data Visualization and Exploration. *Patterns*. 2020; doi: 10.1016/j.patter.2020.100122.

2. Chervitz SA, Deutsch EW, Field D, Parkinson H, Quackenbush J, Rocca-Serra P, et al.. Data Standards for Omics Data: The Basis of Data Sharing and Reuse. In: Mayer B, editor. *Bioinformatics for Omics Data*. Totowa, NJ: Humana Press;

3. Chiang G-T, Clapham P, Qi G, Sale K, Coates G. Implementing a genomic data management system using iRODS in the Wellcome Trust Sanger Institute. *BMC Bioinformatics*. 2011; doi: 10.1186/1471-2105-12-361.

4. Courtot M, Gupta D, Liyanage I, Xu F, Burdett T. BioSamples database: FAIRer samples metadata to accelerate research data management. *Nucleic Acids Research*. 2022; doi: 10.1093/nar/gkab1046.

5. Gray J, Liu DT, Nieto-Santisteban M, Szalay A, DeWitt DJ, Heber G. Scientific data management in the coming decade. *SIGMOD Rec*. 2005; doi: 10.1145/1107499.1107503.

6. Hedges M, Blanke T, Hasan A. Rule-based curation and preservation of data: A data grid approach using iRODS. *Future Generation Computer Systems*. 2009; doi: 10.1016/j.future.2008.10.003.

7. Higgins SG, Nogiwa-Valdez AA, Stevens MM. Considerations for implementing electronic laboratory notebooks in an academic research environment. *Nat Protoc*. 2022; doi: 10.1038/s41596-021-00645-8.

8. Kuhn Cuellar L, Friedrich A, Gabernet G, de la Garza L, Fillinger S, Seyboldt A, et al.. A data management infrastructure for the integration of imaging and omics data in life sciences. *BMC Bioinformatics*. 2022; doi: 10.1186/s12859-022-04584-3.

9. Kuhring M, Nieminen M, Kirwan J, Beule D, Holtgrewe M. AltamISA: a Python API for ISA-Tab files. *JOSS*. 2019; doi: 10.21105/joss.01610.

10. Kunszt P, Blum L, Hullár B, Schmid E, Srebniak A, Wolski W, et al.. iPortal: the swiss grid proteomics portal: Requirements and new features based on experience and usability considerations. *Concurrency Computat: Pract Exper*. 2015; doi: 10.1002/cpe.3294.

11. Mohr C, Friedrich A, Wojnar D, Kenar E, Polatkan AC, Codrea MC, et al.. qPortal: A platform for data-driven biomedical research. Lisacek F, editor. *PLoS ONE*. 2018; doi: 10.1371/journal.pone.0191603.

12. Rambold G, Yilmaz P, Harjes J, Klaster S, Sanz V, Link A, et al.. Meta-omics data and collection objects (MOD-CO): a conceptual schema and data model for processing sample data in meta-omics research. *Database*. 2019; doi: 10.1093/database/baz002.

13. Sansone S-A, Rocca-Serra P, Field D, Maguire E, Taylor C, Hofmann O, et al.. Toward interoperable bioscience data. *Nat Genet* 2012; doi: 10.1038/ng.1054.

14. Sanghi A, Gruber JJ, Metwally A, Jiang L, Reynolds W, Sunwoo J, et al.. Chromatin accessibility associates with protein-RNA correlation in human cancer. *Nat Commun*. 2021; doi: 10.1038/s41467-021-25872-1.

15. Bischof J, Wilke A, Gerlach W, Harrison T, Paczian T, Tang W, et al.. Shock: Active Storage for Multicloud Streaming Data Analysis. *2015 IEEE/ACM 2nd International Symposium on Big Data Computing (BDC)*. Limassol: IEEE;

16. "Ernst M, "Fuhrmann P, "Gasthuber M, "Mkrtchyan T [Deutsches E (Germany)]", "Waldman C [Fermi NAL Batavia, IL (United States)]". dCache, a distributed storage data caching system. China: Science Press, Beijing (China);

17. Sheffield NC, Stolarczyk M, Reuter VP, Rendeiro AF. Linking big biomedical datasets to modular analysis with Portable Encapsulated Projects. *GigaScience*. 2021; doi: 10.1093/gigascience/giab077.

18. Bauch A, Adamczyk I, Buczek P, Elmer F-J, Enimanev K, Glyzewski P, et al.. openBIS: a flexible framework for managing and analyzing complex data in biology research. *BMC Bioinformatics*. 2011; doi: 10.1186/1471-2105-12-468.

19. Nieminen M, Stolpe O, Schumann F, Holtgrewe M, Beule D. SODAR Core: a Django-based framework for scientific data management and analysis web apps. *JOSS*. 2020; doi: 10.21105/joss.01584.

20. Ton Smeele, Chris Smeele. Davrods, an Apache WebDAV interface to iRODS. *Proceeding of iRODS User Group Meeting 2016*. 2016.

21. Holtgrewe M, Stolpe O, Nieminen M, Mundlos S, Knaus A, Kornak U, et al.. VarFish: comprehensive DNA variant analysis for diagnostics and research. *Nucleic Acids Research*. 2020; doi: 10.1093/nar/gkaa241.

22. Stolpe O, Nieminen M, Obermayer, Benedikt, Weiner, January, Beule, Dieter, Holtgrewe, Manuel.

Kiosc: an integrated platform for managing bioinformatics data analysis containers. *Submitted*.

Figure 1

Click here to access/download;Figure;Figure_1.pdf

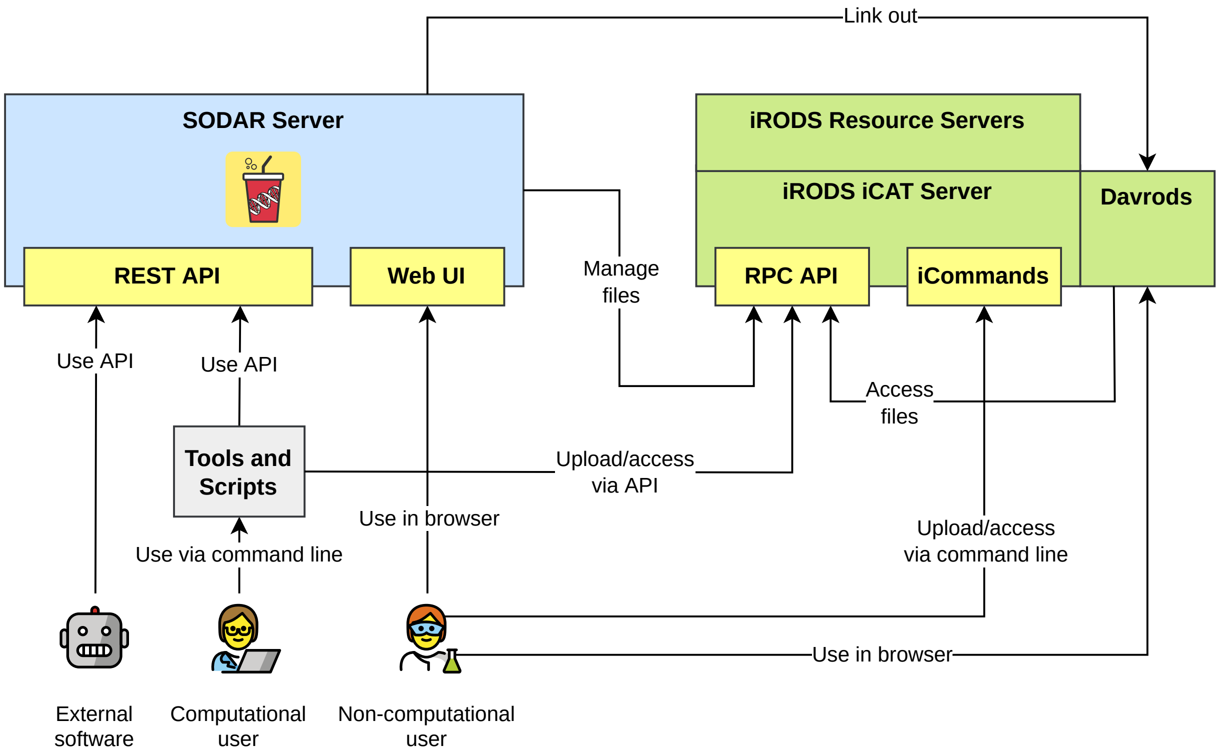

Figure 2

Click here to access/download;Figure;Figure_2.pdf

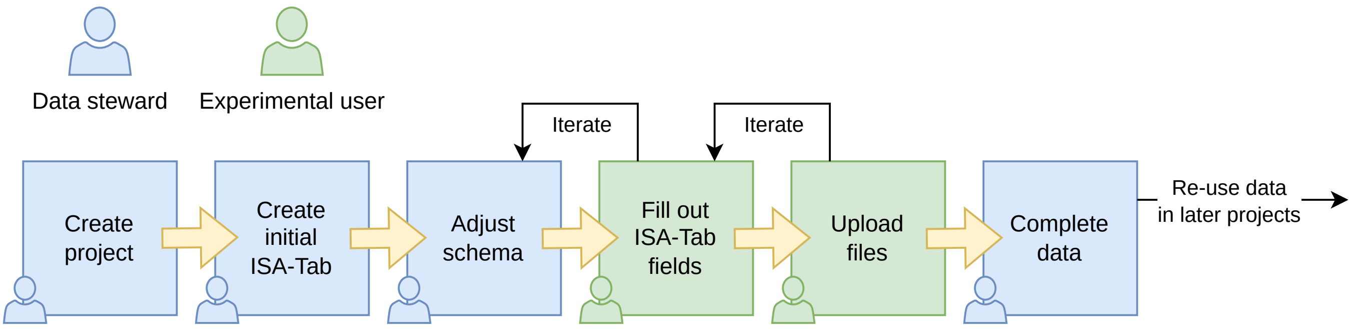

Supplementary Figure 3

Click here to access/download
Supplementary Material
Figure_3_Supplementary_Material.pdf
