# Supplementary material for: SODAR: managing multiomics study data and metadata
Source: Gigascience. 2023 Jul 27;12:giad052. doi: 10.1093/gigascience/giad052 (PMC10373112; doi:10.1093/gigascience/giad052)

# GigaScience


## SODAR: managing multi-omics study data and metadata

--Manuscript Draft--

| | |
|---|---|
| **Manuscript Number:** | GIGA-D-22-00194R1 |
| **Full Title:** | SODAR: managing multi-omics study data and metadata |
| **Article Type:** | Technical Note |
| **Funding Information:** | Bundesministerium für Bildung und Forschung (031L0220A) — Mr. Mikko Nieminen<br>Deutsche Forschungsgemeinschaft (427826188) — Mr. Mikko Nieminen |
| **Abstract:** | Scientists employing omics in life science studies face challenges such as the modeling of multi assay studies, recording of all relevant parameters, and managing many samples with their metadata. They must manage many large files that are the results of the assays or subsequent computation. Users with diverse backgrounds, ranging from computational scientists to wet-lab scientists, have dissimilar needs when it comes to data access, with programmatic interfaces being favored by the former and graphical ones by the latter.<br>We introduce SODAR, the system for omics data access and retrieval. SODAR is a software package that addresses these challenges by providing a web-based graphical user interface for managing multi assay studies and describing them using the ISA (Investigation, Study, Assay) data model and the ISA-Tab file format. Data storage is handled using the iRODS data management system, which handles large quantities of files and substantial amounts of data. SODAR also offers programmable APIs and command line access for metadata and file storage.<br>SODAR supports complex omics integration studies and can be easily installed. The software is written in Python 3 and freely available at https://github.com/bihealth/sodar-server under the MIT license. |
| **Corresponding Author:** | Mikko Nieminen, M.Sc.<br>Berlin Institute of Health at Charite<br>Berlin, Berlin GERMANY |
| **Corresponding Author Secondary Information:** | |
| **Corresponding Author's Institution:** | Berlin Institute of Health at Charite |
| **Corresponding Author's Secondary Institution:** | |
| **First Author:** | Mikko Nieminen, M.Sc. |
| **First Author Secondary Information:** | |
| **Order of Authors:** | Mikko Nieminen, M.Sc. |
| | Oliver Stolpe |
| | Mathias Kuhring |
| | January Weiner |
| | Patrick Pett |
| | |

Powered by Editorial Manager® and ProduXion Manager® from Aries Systems Corporation

| | |
|---|---|
| | Manuel Holtgrewe |
| | Dieter Beule |
| **Order of Authors Secondary Information:** | |
| **Response to Reviewers:** | Dear Hongling Zhou,<br><br>Thank you very for the reviews and for giving us the chance to revise our manuscript. We have carefully considered the comments and answered the questions of both reviewers and improved the manuscript, accordingly, see details below in our answers to the individual reviewers' comments, questions and suggestions. You can find the original response by you and the editors in black below and our answers in red.<br><br>We also thank you for extending the deadline for resubmission. It took us longer to appropriately respond to the reviewers' issues than we hoped for. We have done comprehensive updates to the manuscript and improved the SODAR software for addressing the reviewers' concerns. As a highlight, the manuscript now contains a greatly extended section on data management software categories and comparison. The SODAR software has been also updated to address the reviewers' concerns (a total addition of six thousand lines of code).<br><br>We have also greatly extended the projects in the SODAR demo server.<br><br>------<br><br>Editor:<br><br>The paper may need serious proofreading by an English native speaker, reviewers think it rushed and verbose.<br><br>Answer:<br><br>We have carefully reviewed the resubmitted manuscript for language and reading flow. We ask the reviewers to consider the updated text. If the reviewers think that issues persist, then we will address them subsequently.<br><br>------<br><br>Reviewer #1:<br><br>The authors developed the SODAR tool, which supports multi-omics integration studies. This is a great tool that has a user-friendly interface and supports multi-omics integration. However, I have several concerns that need to be addressed before this manuscript can be considered to be published.<br><br>Answer:<br><br>Thank you for your time and input in writing your review. We think that we have addressed your points in this letter and the manuscript.<br><br>------<br><br>Reviewer #1:<br><br>How does the SODAR handle the multi-omics data that are from different samples? For example, the gut microbiome data from stool samples and proteomics data from blood samples, which may be from the same person but collected at different dates.<br><br>Answer:<br><br>This point is already addressed by the ISA-Tab file format and more a question of data model than of the SODAR system. The example given by the reviewer could be modeled by having the "person" as the ISA source and the different stool samples as ISA sample stemming from the same ISA source. The individual ISA samples can be |

annotated with the date each. Each ISA sample can then be subjected to an individual ISA assay, e.g., one “metagenome” and one “metatranscriptome” assay.

------

Reviewer #1:

Since SODAR supports cell editing, so how does it make the metadata and expression data consistent automatically?

Answer:

The user can update the meta data and create “invalid data” here, e.g., destroy links between the meta data and mass data files. We have updated the SODAR user interface software such that it will notify the user about such broken links such that corrective action can be taken.

Some information about linking meta and mass data can be found in the manual:

- https://sodar-server.readthedocs.io/en/latest/metadata_advanced.html

The sample sheet editor user interface is already keeping the structure of the meta data itself consistent. For example, the editor does not allow removing a source or sample from the “study” table if it is referenced by a sample in an “assay table”. Also, the ISA data model requires us to keep rows redundant with the same information in certain cases. For example, if there is a “split” in processes at some point, the rows left of it relating to the same “split source” material must be equal. The SODAR sample sheet editor ensures such invariants. This way, intrinsic consistency is kept automatically.

A full list of changes to the source code can be found here in the changelog.

- https://sodar-server.readthedocs.io/en/latest/sodar_changelog.html
- https://sodar-core.readthedocs.io/en/latest/changelog.html

------

Reviewer #1:

The authors claim that the SODAR can support multi-omics integration studies. However, I didn't find out how SODAR can do that. Could the authors give more descriptions about that?

Answer:

We have toned down the integration aspect as a response to the second reviewer.

Nevertheless, SODAR supports storing data from multi-omics studies through the ISA data model. For one study, users can store multiple assays, e.g., DNA-seq, RNA-seq, and metaproteomics. Results from each assay can be linked to the same original in the same study.

E.g., there can be the “Person 1” source, the “Person 1 blood” sample as well as “Person 1 stool”. The “Person 1 blood” can then be subjected to DNA and RNA sequencing in individual assays generating “Person 1 blood DNA FASTA” and “Person 1 blood RNA FASTA” files with subsequent analyses (variant calling, RNA expression analysis) while a metaproteomics assay could be performed the “Person 1 stool” sample to yield “Person 1 stool metaproteomics” data.

Some more examples can be found here in the SODAR Server Documentation

- https://sodar-server.readthedocs.io/en/latest/metadata_recording.html?highlight=example#metadata-recording

The meta data of this study can be stored as ISA-Tab in SODAR and the mass data and individual result files stored in SODAR's iRODS server. Users can then retrieve the sample sheet and secondary/tertiaray analysis files from SODAR and integrate them to, hypothetically, correlate genotype with RNA expression and stool metaproteomics results in a Jupyter notebook or R session.

------

Reviewer #2:

The reviewer thanks the authors for their efforts in producing the submitted manuscript.
The authors describe a django based web application designed to support data management.
The tool is built to support experimental metadata capture using the ISA format in its tsv form.
The tool relies on irods to manage data files associated with the experimental metadata.
The tool offers programmatic access via an API and clear front end.

Answer:

Thank you very much for your valuable time and your helpful comments and suggestions. We have improved our manuscript accordingly and addressed issues raised by the other reviewer. Please find details concerning the individual points you raised below.

------

Reviewer #2:

The title: "SODAR: enabling, modeling, and managing multi-omics integration studies" could be clearer. Being more concise "SODAR: standard compliant management of multi-omics studies " would deliver a better message.

Answer:

We have toned down the integration aspect and instead changed the title to the simpler "SODAR: managing multi-omics study-data and metadata" to address your concerns.

------

Reviewer #2:

Page 1 , Abstract: it would benefit from further refinement as there are several repetitions.

Answer:

We have updated the abstract.

------

Reviewer #2:

Check 3rd sentence for English. "ranging from....to..." , s/whereas/to/
"Scientists from diverse backgrounds also have different demands for interfacing with the data, ranging
from computational users that need programmatic or command line access whereas non-computational
users need graphical interfaces."

to:

"Scientists, with different backgrounds, ranging from computational scientists to wet-lab scientists, have different needs when it comes to data access, with programmatic interfaces being favoured by the former and graphical ones by the latter".

Instead of saying "under a permissive licence", be more explicit and plainly state "under MIT licence."

Answer:

We have updated this text.

------

Reviewer #2:

what is the difference between " data analysis and integration of data"?

Answer:

We have reworked this part of the manuscript.

------

Reviewer #2:

Repetition/redundancy in "An example of such complex study is (Esterhuyse et al., 2015) in infection biology, which will be used as an example below."

Answer:

We have removed this running example and particular part of the text.

------

Reviewer #2:

Use of term "modeling": using "plan" or "planning" may be better to remove any ambiguity about the nature of the modeling (statistical modeling, data modeling). Alternating, perfer 'representation' or 'representing'.
(the term model is repeated many times in the following sentences)

Answer:

Thank you for the thoughtful suggestions. We have adjusted and streamlined the terminology. We hope that it is now better/clearer.

------

Reviewer #2:

The statement "The most comprehensive standard for describing study metadata is the ISA-Tab format ..." is probably too strong. There are more formal (UML) models such as FUGE-OM (https://doi.org/10.1038/nbt1347 ) or CDISC SDM & SDTM.

A more understated assessment such as "a popular standard, owing to its simplicity, is the ISA-Tab format"

"Alternatives include..." possibly cite other options for managing such complex datasets as seen with BIDS in neuroscience (Gorgolewski, K., Auer, T., Calhoun, V. et al. The brain imaging data structure, a format for organizing and describing outputs of neuroimaging experiments. Sci Data 3, 160044 (2016). https://doi.org/10.1038/sdata.2016.44) or why not mention HDF5 specification.

Powered by Editorial Manager® and ProduXion Manager® from Aries Systems Corporation

Answer:

We have restructured and rewritten these parts of the manuscript to address your concerns. In particular, we made the wording more “open” in that we do not aim a comprehensive description of available standards but simply mention some relevant ones in our context.

------

Reviewer #2:

This section could be improved by refining the transitions between the different ideas presented or organising the flow.

For example, by layout out the challenges of 1/ dealing with experimental metadata and 2/ dealing with digital objects produced by instruments, which have the characteristics outlined by the authors (volume, depth). Then review the technical solutions and then present the choices made by this implementation and possibly identify the selection criteria which led to choosing one specification over another.

Answer:

Thank you for raising this valid and important issue. We have thought long and hard how to appropriately address this. First, we restructured the text according to your suggestion in the layout.

Second, we have built on the categories by Machina & Wild (2013) to create a possible categorization of available software for management of scientific data. We think that this reflects the different aims and strengths of different software better than the original text and stresses how different aims and purposes shape different strengths in certain areas. We also think that this now explains how different software packages complement each other but also that there are no “hard lines” between features as some are shared between packages from different categories. Some packages attempt to provide comprehensive feature sets while others specialize in certain areas.

------

Reviewer #2:

Page 4: " Non-computational users can interface with SODAR using the graphical UI, whereas computational users can use command line interfaces and REST APIs from scripts and other external software."

Repeat from the abstract. I would suggest rephrasing to 'humanise' 'computational users' vs 'non-computation users', and identifying the function and roles in actual labs (bioinformaticians, data analysts, aka dry lab scientists) vs (experimentalists, wet-lab biologists).

Answer:

It is our opinion that the figure and wording therein is fine. We define dry lab scientist vs wet lab scientist as follows. Dry lab scientists are able to use the command line while wet lab scientists are “limited” to using graphical user interfaces. This distinction is not perfect, but we consider it useful for our purposes.

------

Reviewer #2:

Figure 1: same comment (in fact confirming by the choice of characters).

a question about the diagram: Is it the case that the Web UI does not talk to server via the API as done in some modern development. Probably highlight there the reliance on the Django framework.

Answer:

In the case of SODAR, the Web UI and the server are bundled together in a single Django project. The use of Django has been highlighted in the revised manuscript.

------

Reviewer #2:

Section 2.1
The first sentence needs attention, check the English. "for both serving for modeling experiments..."

Answer:

We have reviewed the manuscript text for this kind of language issues using support form native speakers.

------

Reviewer #2:

Also, there are systems (EBI Metabolights tools on their github repo, DataVerse, FAIRdom SEEK, Zendro...).

So the story telling should probably first talk about the survey of the existing and then only bring to arguments justifying new development.

Answer:

As mentioned above, we have reworked the text and think that the new version properly addresses the raised concerns and gives a proper and "fair" treatment of the considered software packages.

------

Reviewer #2:

Table 1.
It is odd to lump blanket statements for tools such as LIMS, ELN  or 'Study Databases' without clearly stating which ones specifically have been evaluated.

It seems that one could formulate a table with very different results.

Question: How was selection bias controlled for?

Answer:

We have addressed this point together with the previous one. After giving the topic lots of consideration, we do not think that a bias free selection of tools is actually feasible. Finding open source tools through Google or Github is hard. We have made an attempt to make a reasonable choice and think that we expressed this more clearly now in the manuscript.

We think that our solution in defining several "categories" of software packages based on the list of packages from Machina & Wild (2013) and considering important features inside each category gives a more objective treatment of the subject than our previous versions. We are interested in the reviewers' opinion about the new text and would welcome suggestions to improve it further.

------

Reviewer #2:

Powered by Editorial Manager® and ProduXion Manager® from Aries Systems Corporation

Page 5:
This section should be reorganised and each explanatory statement refined to add clarity. Case in point:
"Arbitrary Experiments": Does experiment equate 'ISA.Assay'? is it akin to a Workflow or process Sequence ?

Answer:

Yes, the meaning of “experiment” in our manuscript directly maps to an “ISA.Assay”. There, material/process flow can be expressed directly as directed acyclic graphics while back-references could be expressed informally by providing material/process names by “ISA Comments” or similar.

------

Reviewer #2:

Question: among the key feature that such a system should have to support the work of dry/wet lab scientists, surely, deposition to public repositories should be high on the list. Why is this absent?

Answer:

While there is no implementation of such a functionality from the WebUI, automatized export is of course possible using the API and in fact, in some projects, this is precisely how we do it. However, as SODAR does not focus on one data type, submission to public repositories is project specific. While some automatization is possible, in our experience publishing data requires often manual work.

We have added text to the manuscript clarifying this.

------

Reviewer #2:

Page 6:
typo: s/bioinfsormaticians/bioinformaticians/
punctuation: to be checked: missing commas make for a difficult read.
suggestion: simplify the role of 'experimentalists' in the context of SOBAR.
"They use the templates provided by the Data Stewards to instantiate a wet lab track and track its metadata."

Answer:

We have incorporated these language/wording issues.

------

Reviewer #2:

Question: How are data stewards trained in ISA-Tab?

Answer:

We have produced internal training material for data stewardship within our organization and projects. However, we consider this outside of the scope of this manuscript.

------

Reviewer #2:

Access to the demo tool gives the opportunity to use and test the component. While

| | |
|---|---|
| | the UI is simple and intuitive, a number of limitations in the editing functionality make usage more difficult that it needs to be.<br><br>Answer:<br><br>The current set of supported features in the editor is based on tight collaboration with the 350+ users of our SODAR implementation. Limiting the number of features is a design choice to make the interface simpler, more intuitive and user-friendly, and we tried to pick these features which are commonly needed by our users. Of course, we will implement new features when the need arises.<br><br>------<br><br>Reviewer #2:<br><br>Page 7:<br>"of course, using the REST-API of SODAR, it is possible to automate these tasks"<br>Could the author produce a jupyter notebook showing how to do so?<br>It would be a nice addition and possibly a good resource that could facilitate uptake.<br><br>Answer:<br><br>We have added examples of API use into the SODAR Server manual here:<br>https://sodar-server.readthedocs.io/en/dev/api_examples.html<br><br>These examples can also be used in a notebook or scripts to serve as basis for task automation in SODAR.<br><br>------<br><br>Reviewer #2:<br><br>Section 2-3:<br><br>page 8-9-10: this section could be streamlined and condensed to really focus on the interaction between shaping a sample processing & data acquisition workflow into a template which can be used by a wet lab scientists.<br>All this while allowing a markup with ontology terms.<br><br>Note: the ontology terms on the demo server do not resolve properly.<br><br>Answer:<br><br>Thank you for the remark, we have fixed this. The ontology term import to the demo server had failed earlier.<br><br>------<br><br>Reviewer #2:<br><br>Question: Why choosing  Bioportal over other services, e.g. EBI OLS?<br><br>Answer:<br><br>There is no particular reason and we are/were not aware of any advantages or issue with either.<br><br>------<br><br>Reviewer #2:<br><br>Question: How can value-sets be constrained in SODAR?<br><br>Answer: |

It is currently possible to constrain sets for simple string/numerical values, but not for ontology terms. A future upgrade to enable specifying allowed ontology term sets has been planned and can be found as issue #1615 in the sodar-server repository issue tracker.

------

Reviewer #2:

Question: ontology browser: it is unclear if the ontologies need to be loaded locally or if they are accessed via an API call to the relevant services ?  Can the authors clarify this point?

Answer:

Ontology terms can be manually set, but for automated lookup in the UI, uploading ontologies into the system is required with system administrator capabilities.

------

Reviewer #2:

the demo server did not seem to allow it or I wasn't able. may be a figure showing the functionality would help?

Answer:

Local upload is done using the UI or management commands, but this functionality is limited to administrators.

------

Reviewer #2:

Page 11: Internal Usage Statistics
Question: it seems that the mean size of an experiment stored in SODAR is ~60 samples and about 10 files per sample.
These are relatively small sized studies.
Can the authors provide insights about the performance of the platform with large studies (several thousands of samples and above) ?

Answer:

We have added a figure showing number of files vs. Total file size for the projects. This illustrates that we have some large studies in our internal instance already (50+TB, 60+k files). The iRODS system can scale to millions of files overall, e.g., the Sanger instance stores thousands of Illumina sequencing runs with tens of thousands of files each with several PB of data.

Further, we have created a large sample project in the demo site with generated “mock” data that shows that the meta data system can handle thousands of samples.

------

Reviewer #2:

Question: Installation and deployment of SODAR.
Why the authors omit to mention that SODAR can be deployed via Docker? It seems useful information.

Answer:

This is mentioned in chapter 3.5, “SODAR Administration”.

------

Reviewer #2:

Question: AltamISA
Checking the library, it seems that development has stalled. It is a concern ?

Answer:

The library is developed by us SODAR authors and we keep it updated as we find identify limitations and bugs. The test coverage is at 95% and so far, we have encountered relatively few bugs per year. We consider the library to fit our scope very well and consider it stable and maintain it in case of bugs.

------

Reviewer #2:

Have the authors tested swapping AltamISA with ISA-API ?

Answer:

When we started out with ISA support in 2018, we found several concerns with ISA-API (we even started to make some fixes at https://github.com/mkuhring/isa-api/tree/cubi-hotfixes). One important feature was “round-tripping”, I.e., loading a file, writing it out again and getting more or less the same content. We checked on 2022-09-13 and the current main branch does not have working CI. Furthermore, a self-written library is much easier to extend for features such as extended validation. ISA-Tab files can be exported from SODAR and used with ISA-API.

------

Reviewer #2:

Is it at all possible ? could it be made via an adaptor of some sort ?

Answer:

This is not currently planned. However, if the need arises, it is possible to develop support in the future or integrate a pull request from a third party.

------

Reviewer #2:

Can Altam ISA convert to ISA-JSON or other public repository compatible format to provide a capability to assist users disseminate their results?

Answer:

We have not yet implemented ISA-JSON support for Altamisa as we have not found the need for it. So far, we have not found any adopters of ISA-JSON besides the ISA-JSON authors but would be interested to learn about them.

SODAR currently focuses on storing studies while they are running. In our use case within a clinic environment, many projects’ data cannot be deposited in public databases, in particular given our background in the German regulatory system. When contributing to public databases, we have written custom scripts to prepare GEO submissions, for example. As SODAR offers REST APIs and allows to export ISA-Tab we have found this sufficient.

------

| | |
|---|---|
| | Reviewer #2:<br><br>figure 3 should not be a supplementary material but a proper content as it is useful as showcasing SODAR UI and customization.<br><br>Answer:<br><br>As we understand it, including such a large figure should be done as a supplement according to this journal's conventions. We believe it is best to leave this to the editor to decide. |
| **Additional Information:** | |
| **Question** | **Response** |
| Are you submitting this manuscript to a special series or article collection? | No |
| **Experimental design and statistics**<br><br>Full details of the experimental design and statistical methods used should be given in the Methods section, as detailed in our Minimum Standards Reporting Checklist. Information essential to interpreting the data presented should be made available in the figure legends.<br><br>Have you included all the information requested in your manuscript? | Yes |
| **Resources**<br><br>A description of all resources used, including antibodies, cell lines, animals and software tools, with enough information to allow them to be uniquely identified, should be included in the Methods section. Authors are strongly encouraged to cite Research Resource Identifiers (RRIDs) for antibodies, model organisms and tools, where possible.<br><br>Have you included the information requested as detailed in our Minimum Standards Reporting Checklist? | Yes |
| **Availability of data and materials**<br><br>All datasets and code on which the | Yes |

| | |
|---|---|
| conclusions of the paper rely must be either included in your submission or deposited in publicly available repositories (where available and ethically appropriate), referencing such data using a unique identifier in the references and in the “Availability of Data and Materials” section of your manuscript.<br><br>Have you have met the above requirement as detailed in our Minimum Standards Reporting Checklist? | |

Powered by Editorial Manager® and ProduXion Manager® from Aries Systems Corporation

Manuscript
Click here to access/download;Manuscript;2022_Nieminen_SODAR_Manuscri

# SODAR: managing multi-omics study data and metadata

Mikko Nieminen[1], Oliver Stolpe[1], Mathias Kuhring [1], January Weiner 3rd [1], Patrick Pett[1], Dieter Beule[*,1], and Manuel Holtgrewe [*, 1]

[1]Berlin Institute of Health at Charité – Universitätsmedizin Berlin, Core Unit Bioinformatics (CUBI), Charitéplatz 1, 10117 Berlin, Germany

[*]These authors contributed equally

## Abstract

Scientists employing omics in life science studies face challenges such as the modeling of multi assay studies, recording of all relevant parameters, and managing many samples with their metadata. They must manage many large files that are the results of the assays or subsequent computation. Users with diverse backgrounds, ranging from computational scientists to wet-lab scientists, have dissimilar needs when it comes to data access, with programmatic interfaces being favored by the former and graphical ones by the latter.

We introduce SODAR, the system for omics data access and retrieval. SODAR is a software package that addresses these challenges by providing a web-based graphical user interface for managing multi assay studies and describing them using the ISA (Investigation, Study, Assay) data model and the ISA-Tab file format. Data storage is handled using the iRODS data management system, which handles large quantities of files and substantial amounts of data. SODAR also offers programmable APIs and command line access for metadata and file storage.

SODAR supports complex omics integration studies and can be easily installed. The software is written in Python 3 and freely available at https://github.com/bihealth/sodar-server under the MIT license.

**Keywords:** Scientific Data Management, ISA-Tab, iRODS

## 1. Introduction

Modern studies in life sciences rely on "omics" assays, which encompass branches of science such as genomics, proteomics, and metabolomics. One or multiple assays can be run within a single study, potentially including assays for multiple omics studies of several types.

The following key steps are required for executing these complex omics studies: a) planning which results in study metadata; b) collection of mass data; and c) data analysis, including the integration of multiple assays. The aim of SODAR is to ensure support for scientists within all the steps.

### *1.1 Challenges*

Each step presents its own set of challenges. During planning it is important to enable recording crucial factors and covariates. The flow of materials and samples through processes must also be specified in sufficient detail. Further challenges arise from, e.g., assays using complex multiplexing, such as the need for reference samples; requirements for using controlled vocabularies or ontologies; and possible change of assays over time.

In the data collection step, scientists must record the used machines, kits, and versions of both hardware and software used. Omics studies also create large volumes of data, ranging from a few gigabytes for mass spectrometry to terabytes for imaging such as microscopy. This data may be spread among many files, further complicating the needs for managing mass data storage. Instead of a rigid

process, data collection should also be adjustable to changes and developments in data generation over time.

Data analysis is often split into multiple phases, with primary analysis of each assay followed by steps for integration of results. Specific results need to be fed back to metadata management, annotation, quality control or storing resulting markers. Access to metadata with recorded factors and confounders is necessary in each step, while access to primary raw data becomes less important after the primary analysis. Certain analysis results are written back into the mass data storage. This includes binary alignment map (BAM) files, and variant call format (VCF) files.

There are also overarching challenges for the steps in study execution. All data should be recorded in structured format. Automation should be applied where possible, and on-premise installation might be preferable or even required when data privacy relevant data is generated such as DNA sequencing.

### *1.2 Data Management Approaches*

In this and the following section, we will discuss the topic of data management and software. The terms “data” and “document” will be used interchangeably in this section. The steps described in section 1.1 can be interpreted as processes taking documents and materials as input, and generating more documents and materials as the result. For example, data collection takes the plan document and samples and generates assay result files (documents). Scientists thus need computational tools for supporting them in managing their scientific and research data.

Historically, such documents are maintained on paper in laboratory notebooks, or documentation created by quality control systems. For the most direct and unstructured approaches in maintaining digital data, this corresponds to word processing, spreadsheet, and image files on local or network

drives. More structured approaches are desireable for taking advantage of digital documents, preventing research data loss [1] or fostering re-use [2].

While data management in science is a broad topic, the library and information science community is frequently approaching itusing a top-down approach. Frequently, in this context, the term “research data management” (RDM) is used. Here, the needs of whole organizations and their parts for managing their resarch data, as well as the necessary steps to establish whole RDM systems are considered first, for example cf. Donner [3]. This correlates with the role of libraries in certain academic organizations for organizing data that was collected in research.

A second approach which can be described as “bottom-up” originates from different “working scientist” communities. The communities commonly refer to the topic as “scientific data management” (SDM) and solve their problems at hand, often starting with specific small-scale solutions which are then upscaled if the need arises. While considering their organizational embedding, they focus on solving specific data management challenges for themselves and their peers. We found ourselves in this situation and will thus focus on this perspective.

### *1.3 Data Management Software Packages*

Scientific data management needs come in different forms and shapes. We could find no general treatment of the subject of data management in the literature. Machina and Wild [4] provide a collection of four tool categories: laboratory information management systems (LIMS), electronic laboratory notebooks (ELN), scientific data management systems (SDMS) and a chromatography data system that we generalize as instrument-specific data system (IDS). In this section, we provide our take on explaining what these systems comprise. We also note – as Machina and Wild [4] did – that categorization of such software solutions is not clear-cut, and features may be overlapping. We expand

this list by two more system types: data repository systems (DRS) and database/data warehouse management frameworks (DMF).

The four items by Machina and Wild [4] are as follows:

**LIMSs** focus on storing information around laboratory workflows. This includes tracking of consumables, samples, instruments, and tests. They deal with daily tasks of laboratories such as billing and instrument calibration. They are often specific to certain domain areas such as sequencing facilities.

**ELNs** focus on allowing humans to record their laboratory work. They replace paper notebooks and capture experiments and their results, mostly in free-form text, pictures, tables etc. They play a key role in fulfilling regulatory requirements.

**IDSs** provide data capturing, storage, and analysis functionality in instrument-specific domains. Two examples are the CASAVA pipeline and the BaseSpace cloud-based service, both from Illumina. The former is provided without extra cost with the instrument along with its source code, while the latter is purchasable and closed source. Such software often ships with the instruments themselves.

**SDMSs** provide scientific content management functionality for scientific data and documentation. They allow for the management of metadata and potentially mass data. Their core functionality does not include data analysis, user-centric data collection, or laboratory workflow tracking. Such features may be potentially supported by plugins or extensions. Many such systems offer integration with surrounding systems, e.g., via application programming interfaces (APIs).

We augment this list by two system types:

**DRSs** provide shared access to data with appropriate documentation and metadata. Examples are FAIRdom Seek [5], Dataverse [6], and Yoda [7]. There also specialized DRS focusing on particular use

cases such as dbGAP [8], MetaboLights [9], and Gene Expression Omnibus [10], that allow for managing public or controlled public access to large research data collections.

**DMFs** allow for the rapid development of database and data warehouse applications. They often provide pre-existing components to build on readymade functionality and extension by implementing custom components. Such enable creating domain-specific databases and structured data capturing. Examples include Molgenis [11] and Zendro [12].

Other types of systems also exist and not every system falls into just one category. A complete review of such systems is beyond the scope of this manuscript. This section identifies focus areas of systems involved in some form of scientific data management. SODAR falls into the category of SDMS.

### *1.4 Data Management Technologies*

For planning and documenting experiments and their structure, experiment oriented metadata storage formats with predefined syntax and semantics exist. A popular standard is the ISA (Investigation, Study, Assay) model [13], which allows describing studies with multiple samples and assays. The ISA model defines the ISA-Tab tabular file format, which allows users to model each processing step with each intermediate result and annotate each of these with arbitrary metadata. An example of an alternative to ISA-Tab is Portable Encapsulated Projects (PEP) [14]. There are also more specialized standards such as Brain Imaging Data Structure (BIDS) for brain imaging data [15], as well as other approaches such as Clinical Data Interchange Standards Consortium (CDISC) standards [16], and the Hierarchical Data Format (HDF5) [17]. Use of generic file formats such as HDF5, TSV, XML and JSON is also common.

For storing large volumes of omics data, it is possible to simply use file systems or object storage systems. More advanced solutions such as Shock [18] or dCache [19] allow for storing metadata and

distributing data over multiple servers. iRODS (Integrated Rule-Oriented Data System) [20, 21] adds further features, such as running rules and programs within the data system and enabling integration with arbitrary authentication methods.

For publication, raw and processed data and metadata are deposited in scientific catalogues, study databases and registries. An example is the BioSamples database for metadata [22].

### *1.5 Our Work*

In our work, we focus on managing many omics projects of varying data size and various use cases including cancer and functional genomics studies. We also need to support multiple technologies such as whole genome sequencing, single cell sequencing, proteomics, and mass spectrometry. Our work is representative of the work typically done by core units in clinics. Clinical settings often deal with humans as their primary sample source. This implies controlled access of data, or not being allowed to share confidential data. Thus, developing support for hosting data in a public repository is not our aim. Likewise, uploading data to other public repositories has not been a priority. Despite the focus of our own projects, the aim is to enable users to adjust the software functionality by supporting flexible forming of study metadata. Open source software is a requirement to avoid vendor lock-in and allow for flexibility in different use cases. A suitable end-to-end solution was not available when we started our work in 2016. Therefore, we set out to implement an integrated system for managing omics-specific data and metadata.

In this manuscript, we introduce SODAR (the System for Omics Data Access and Retrieval). SODAR combines the modeling of studies and assays using the ISA-Tab format with handling of mass data storage using iRODS. More example projects are available in the SODAR online demo at https://sodar-demo.cubi.bihealth.org.

## 2. Results

We present the results by first giving an overview of the developed SODAR system. Next, we compare it to a selection of existing tools and their relevant features. We then describe processes we have established around SODAR. Finally, internal usage statistics are detailed along with discussion on the limitations of SODAR.

### 2.1 Resulting System Overview

Figure 1 presents the components of the SODAR system. The SODAR server is built on the Django web framework. It contains the main system logic and provides both a graphical user interface (GUI) and application programming interfaces (API) for managing projects, studies, and data.

Project and study metadata are stored in a PostgreSQL database. The study metadata is stored as ISA-Tab compatible sample sheets, with each project containing a single ISA-Tab investigation. Each investigation can hold multiple studies, likewise each study can contain multiple assays.

Mass data storage is implemented using iRODS and accessed via iRODS command line tools or access to the WebDAV protocol, which is provided by using the Davrods software. The SODAR server manages creation of expected iRODS collections (i.e., directories), governs file access and enforces rules for file uploads and consistency. Investigations, studies, and assays correspond to collections in the iRODS file hierarchy. Within assays, collection structure can be split by, e.g., samples or libraries, depending on the type of assay.

Uploading files for studies is handled using "landing zones", which are user specific collections with read and write access. The SODAR server handles validation and transfer of files from the landing zones into the project specific read-only sample repository, which is split into assay specific iRODS collections.

Planning and tracking the study design and experiments is done using the ISA-Tab compatible sample sheets. Here, the “assay” in the ISA model corresponds to an “experiment” in our work. SODAR provides multiple ways to create and edit both the metadata model and the contained metadata itself, including user friendly GUI-based creation of sample sheets from ISA-Tab templates. The templates aid in maintaining consistent metadata structures between studies. Once created, the SODAR server provides a graphical UI for filling up metadata and configuring expected values, including support for controlled vocabularies and ontologies. Furthermore, SODAR also allows uploading and updating sample sheets using its API. Uploading any valid ISA-Tab file and replacing existing sheets via upload is also supported, enabling the creation of sample sheets using other software such as ISA-tools [13]. The API allows to automate metadata and file management activities using scripts.

**Figure 1** SODAR system with its components and actors. The figure illustrates how actors interact with SODAR and iRODS through different APIs.

*2.2 Data Management Software Features and Selection*

This section first describes features of DMS packages that are subsequently used for comparing SODAR to other software types and packages. We then describe the selection process for software comparison.

The following is a list of features that allows us to see the unique strengths and properties of SODAR in the category SDMS and describe the difference to other categories. When a feature is important in multiple categories, it is only shown once. Categories 1-4 are focused on SDMS, and category 5 contains features also important for other categories.

1) Features addressing overarching challenges

a) Structure into projects and folders

b) Access control

c) Automation possible via API

2) Use of open formats and standards

a) Features addressing planning challenges

b) Structured recording of assays and experiments

c) Flexibility in definition of studies and experiments

d) Annotation with controlled vocabulary

e) Annotation with ontologies

3) Features addressing data collection challenges

a) Storage of files possible

b) Support for many files

c) Support for large file sizes

4) Features addressing data analysis challenges

a) API for reading and updating experiment metadata

b) API for reading and updating mass data

5) Features commonly found in specific systems

a) ELN

i) Flexible data entry in free text / tables / pictures

b) DRS

i) Host public data repositories

c) DMF

i) Easy creation of new data tables

ii) User-centric data entry

iii) Multiple predefined components, e.g., for data visualization and analysis

With the aim of showing the unique strengths of software categories and packages, we attempted to select popular software packages in each category. We limited the selection to open-source software. We searched for the different software types via a publication on Google Scholar or the project search on GitHub. We made no attempt to define "the most popular" or "the best" software packages. We excluded LIMS and IDS as such software is focused on the wet-lab process. The following software was selected:

1) SDMS
   a) SODAR
   b) qPortal [23]
   c) FAIRDom Seek [5]
   d) OpenBIS ELN-LIMS [24]
2) ELN
   a) ELabFTW [25]
3) DRS
   a) Dataverse [6]
   b) Yoda [7]
4) DMF
   a) Molgenis [11]
   b) Zendro [12]

*2.3 Data Management Software Comparison*

The table included in [Additional File 1] shows the comparison of the categorized software in the categories as described in section 2.2.

Since the software packages operate in a similar space, there is a certain overlap in features, even across categories. Most software packages provide the features for addressing the overarching challenges. All “planning” features are included in SODAR and FAIRDom Seek in the SDMS category, while qPortal and OpenBIS remain limited. ELabFTW provides limited functionality for structured recording and does not support controlled vocabularies and ontologies, while DRS systems do not address planning challenges by their design. As expected, such features can be implemented by the DMF packages, but they do not provide the functionality on their own. The “data collection” and “data analysis” features are only comprehensively addressed by SODAR and FAIRDom Seek in the SDMS category, with FAIRDom Seek being limited in storing many and/or large files. ELN software is limited in this capability, while DRS packages provided good support for such features, and the DMF software packages allow for implementing support to varying levels.

As for the specialized features, some functionalities of “foreign” categories are implemented. For example, SODAR has support for user centric data entry, and FAIRDom Seek allows for hosting public data repositories by design. However, each software package shows its strengths by providing the features for the tasks that it was originally designed for. We note that certain packages cover their category more focused or comprehensively than others. For example, in the DMF category, Molgenis has an ecosystem of many predefined components, while Zendro focuses on allowing for the easy creation of tables and creating user centric data entry masks.

### *2.4 Roles and Interaction with SODAR*

The general workflow in using SODAR for managing data and metadata is shown in Figure 2. We distinguish between the roles “data steward” and “experimentalist.” It is possible for one person to act in both roles.

Data stewards are responsible for creating the overall structure of the experiment data. They are expected to be experienced with using ISA-Tab files. For example, in our use case, data stewards are bioinformaticians working in the core unit. They are responsible for planning the experiments and modeling them in the ISA-Tab format as sample sheets describing the overall experimental design. Data stewards also maintain a library of sample sheet templates for common use cases. With experienced experimentalists the steward might just create the general structure of the experiment. In some cases, the steward may also pre-create the sample sheet with an initial structure of all planned samples and processes and IDs together with experimentalists.

Experimentalists are primarily responsible for entering the actual data into the system. They are users more concerned with completing the metadata in the sample sheet than in creating its structure. When the full sample sheet is created together with data stewards, experimentalists may only verify the structure against the information of their experiments and fill in some measurements in sample sheet cells, e.g., concentration measurements. More experienced experimentalists will also create new rows in the ISA-Tab tables for samples, related materials, and processes.

**Figure 2** SODAR metadata management workflow. The workflow scheme is divided into steps attributed to a data steward (blue) who manages the overall data schema and experimental user (green) who enters the actual data or uploads files.

*2.5 General SODAR Process*

Here we describe the SODAR-backed process of managing experiment data we are using in our work. This demonstrates how SODAR helps tackle challenges in complex omics study management.

*2.5.1 Planning and Sample Sheet Creation*

Planning begins with data steward and experimentalists meeting and discussing the study, including, e.g., its factors, sample size, replicas, and confounders. Stewards create sample sheets from templates and modify columns depending on the discussions and the study's requirements. Working together, stewards and experimentalists also decide on ontologies and controlled vocabularies to use, data ranges, etc.

The template will be bootstrapped with example samples, or all samples, depending on the study. During this step, the experimentalist receives training in using the SODAR sample sheet editor for filling in cells where necessary. Filling cells can involve, e.g., adding measurements, cancer staging, definition and refinement of phenotypes, adjustment of relationship information.

Automated extraction of measurements from instruments or LIMS and ingesting it using the SODAR API is also possible. for example, an integration with a LIMS system could automatically create samples as they are processed in the wet lab, while measurements could be written to SODAR from the LIMS or from an integration of an ELN system. We are currently working towards this when cooperating with other units.

*2.5.2 Data Acquisition and Sample Sheet Update*

Experimentalists run their experiments and use SODAR for editing the sample sheets. This includes adding new samples, marking dropouts, or removing them, and adjusting ontologies and terms as

needed. SODAR sample sheets are useful as a central storage of metadata, removing the need to, e.g., share spreadsheets via email. Differences between sample sheet versions can also be browsed in the SODAR UI to track changes in the metadata.

In this step, actual data files are uploaded by experimentalists to the project sample repository through landing zones. The iRODS collection structure for each study is maintained by SODAR and based on the study type and names of samples or associated libraries. In most cases, files related to a certain sample and its processing in an assay can be found in the collection named after the related library.

*2.5.3 Data Analysis*

For data analysis, bioinformaticians access metadata in the sample sheets as well as raw data in iRODS, the latter being linked to former in the SODAR GUI for ease of access. Depending on the phase of study, this may involve, e.g., primary analysis, secondary analysis, and required data integration. Resulting files are uploaded back into iRODS via SODAR for safekeeping and sharing between researchers. Also uploaded are files needed for integrating with third party systems, such as UCSC Genome Browser [26] tracks and files for data exploration tools such as SCelVis [27].

During the analysis, up-to-date experiment structure is maintained in SODAR. It represents a centralized storage and sole source of truth for the internal structure, encompassing factor values, ontologies, and controlled vocabularies. Similarly, it represents an external structure, with samples and materials linked to corresponding iRODS collections.

SODAR also provides integrations to specific third-party software to aid analysis. For germline and cancer DNA sequencing experiments, SODAR supports the IGV Genome Browser [28], by generating session files pointing at relevant variant and read alignment files with a single click.

*2.5.4 Long-Term Data Storage and Data Access*

After transferring files from landing zones into the project's sample repository, the data is in general assumed to be permanent and not modifiable or rewritable, with users only having the possibility of request file deletion from project maintainer in case of, e.g., mistakes in uploading. Hence, once the project finishes, the data is considered good for long term archival. SODAR supports setting projects into a read-only "archived" state and provides an API for implementing custom policies for handling archived data. For example, such a policy might consist of adding a cold storage resource such as tape onto which the data could be moved.

In exporting data to public databases, creating a generic exporter cannot be considered feasible due to the metadata model flexibility in SODAR. However, there are export possibilities depending on the type of study. For example, if the project is set up with Gene Expression Omnibus (GEO) [10] compatible metadata, exporting to the GEO database may be trivial depending on the target system APIs. In the future, we intend to create export functionality from SODAR to the emerging German National Research Data Infrastructure (NFDI), the associated German Human Genome-phenome Archive (GHGA) [29], and corresponding metadata models. These will be based on the federated European Genome-phenome Archive (EGA) [30] and should provide a good starting point for many other exporters. NFDI will be our long term and controlled public access backend, while other users and instances might have other backends.

*2.6 Internal Usage Statistics*

We have been using SODAR in our group’s projects for the past four years. Table 2 summarizes data statistics and metadata stored in our internal instance and the diversity of projects. We have thus tested

SODAR extensively in a real-world setting and use it daily as our main storage for all our project data and metadata.

**Table 2** Summary statistics of project type and count, sample count, user count, mass data file count and total size in our internal instance of SODAR.

| | |
|---|---|
| **Projects** | 406 |
| **Users** | 385 |
| **Samples** | 26 349 |
| **Total File Count** | 304 638 |
| **Total File Size** | 457 TB |

Statistics collected in March 2023

Figure 3 displays file size and count for each project on our system in March 2022. The diagram shows the varying scale of the projects within our group. A limited number of projects between a 20-45 terabyte range can be seen, while most are smaller.

**Figure 3** SODAR project file statistics scatter plot, with file count per project on the X axis, and the total file size in terabytes on the Y axis.

*2.7 Limitations*

Currently, SODAR offers no automated data export to, e.g., the GEO database. This may be added in the future as discussed in the “Long-term data storage” section. Similarly, SODAR does not support access in a "data commons" manner. It is possible to set specific projects for public read access, but by default SODAR enforces strict access control to data.

We also do not have a definitive solution for training people in ISA-Tab. SODAR features a set of templates for predefined study types for, e.g., germline and cancer studies, but there is no definite solution for trivially setting up any type of study as ISA-Tab.

## 3. Methods

SODAR is implemented in Python 3 using the Django web framework and Django REST Framework. Reusable components have been extracted into the library SODAR Core [31]. ISA-Tab format manipulation has been implemented using AltamISA [32].

### *3.1 Project Organization, Authorization Structure, and LDAP Integration*

SODAR uses the concept of “projects” for organizing all data. Projects have a unique identifier and some basic metadata, such as title and description. Projects are organized in a tree structure using the concept of “categories” that can contain projects or other categories. Each project has a single owner, who can assign themselves a delegate for managing the project. Further users can be granted access to the project either in a read-write (contributor) or a read-only fashion (guest) using Role-Based Access Control (RBAC) [33].

SODAR can be configured to be run standalone or integrated with LDAP servers, including Microsoft ActiveDirectory, for providing authentication information. Here, authentication refers to checking the identity of a user based on their username and password.

### *3.2 iRODS integration*

SODAR automatically manages user access to projects in iRODS. This is done by creating an iRODS directory and user group for each project. The group is given access to the directory and group membership is synchronized between the SODAR database and iRODS.

SODAR creates an iRODS collection for each study and assay from the ISA model of the project. Files can be uploaded by users through landing zones, either for each sample or for the whole study or assay. It is thus possible to add data for an arbitrary number of assays for each sample and original donor or specimen.

The files can be accessed either directly through iRODS or using the WebDAV protocol through the Davrods [34] software. The latter allows users to access the storage as a network drive on their desktop computers. Since WebDAV is HTTP based, users can also make data available to genome browsers such as IGV or UCSC Genome Browser. Moreover, it is easy to access data through an organization's security system and proxies without the intervention of IT departments.

Optionally, SODAR allows the management of iRODS "tickets," which allow for access based on randomly generated tokens instead of user login. This way, users can upload genome browser tracks to SODAR and iRODS and create public URL strings to access them and share them with users that do not have access to the full project, or do not even have an account in SODAR.

### *3.3 Sample Sheet Editor, Import, Export*

Sample sheets can be included into SODAR projects by either importing existing ISA-Tab files or template-based creation. When importing, the user can upload a Zip archive or a set of individual ISA-Tab files. For creating sample sheets from templates, the user needs to fill in certain details in the SODAR GUI. SODAR contains multiple built-in templates for, e.g., generic RNA sequencing, germline DNA sequencing and mass spectrometry-based metabolomics. After import or creation, the sample sheets are stored in an object-based format in the SODAR database for easy search and modification. In the GUI, they are presented to the user as spreadsheet-style study and assay tables.

The user can edit sample sheets in the SODAR GUI [Additional File 2]. Cells in the study and assay tables can be edited like in a spreadsheet application. For each column, the project owner or delegate can define the accepted format, value choices, value ranges, regular expressions for accepted values, and other settings depending on the column type. This ensures the validity of data and its compatibility with the study's requirements and conventions.

SODAR supports ontology term lookup for cell editing. Commonly used ontologies such as Human Phenotype Ontology (HPO) [35], Online Mendelian Inheritance in Man (OMIM) [36], and NCBI Taxonomy Database Ontology (NCBITaxon) [37] can be uploaded into SODAR for local querying as OBO or OWL files, without the need to rely on third party APIs. Manual entering of ontology terms is also allowed. It is possible to include multiple ontology terms in a single cell and one or several ontologies can be used in a single column.

In addition to cell editing, the user can insert and remove rows for study and assay tables. Cells for existing sources, samples, materials, or processes are auto filled by the editor when including a new row. Similarly, if multiple rows contain references to the same entity, all related cells are automatically updated in the tables when modifying them on a single row. SODAR validates all edits using the AltamISA parser [32]. This ensures the validity and ISA-Tab compatibility of the sample sheets at each point of editing.

When editing sample sheets, old sheet versions are stored as backup. These versions can be compared and restored in case of mistakes, as well as exported from the system. SODAR allows for sample sheet export in the full ISA-Tab TSV format, or simplified Excel tables. Replacing existing sheets with versions modified outside of SODAR is also supported.

*3.4 Integrating SODAR Core based sites*

Several subcomponents of the SODAR server such as project and user management have proven to be useful in other contexts. We have extracted them into the SODAR Core software package [31] which forms the foundation of other projects such as VarFish [38] and Kiosc [39]. Using a common library for projects and access management has several advantages and enables the integration of VarFish and Kiosc with SODAR.

SODAR can be configured to work as a “source” site. Applications based on SODAR Core can then be configured as “target” sites of the source site. Projects and access to users will then be synchronized to target sites. This allows us to manage sample and experiment definitions in SODAR and upload corresponding variant data to VarFish. VarFish can then use the REST APIs defined by SODAR for synchronizing sample metadata, such as phenotype terms, directly from SODAR. Similarly, users can upload mass data files into the iRODS data repository and create access tokens to them in SODAR. These tokens can be used to provide data visualization applications in Kiosc with data access via HTTP and iRODS protocols or external applications such as UCSC Genome Browser.

### *3.5 SODAR Administration*

We provide a straightforward way to install SODAR and related components (SODAR, iRODS, Davrods, and supporting database servers) and maintain such an installation based on Docker containers and Docker compose. Detailed installation instructions can be found in the “sodar-docker-compose" repository linked to in the section “4.2 Source Code Availability.”

The entire system can be set up using an external LDAP or ActiveDirectory server for users and credentials, or as an alternative in a standalone fashion where SODAR hosts this information. Existing iRODS installations can also be used with SODAR. For administrators, SODAR features dashboards which provide statistics regarding projects and usage of storage resources.

## 4. Data and Source Code Availability

### *4.1 Data Availability*

A demonstration instance with data is available at https://sodar-demo.cubi.bihealth.org. All source code is available at https://github.com/bihealth/sodar-server. Example metadata for demonstration projects is available in ISA-tab format at https://github.com/bihealth/sodar-paper.

### *4.2 Source Code Availability*

**Project name:** sodar-server

Project home page: https://github.com/bihealth/sodar-server

Operating system: Linux/Unix

Programming language: Python

License: MIT

RRID: SCR_022175

Biotools: biotools:sodar

## 5. Additional Material

Additional File 1

- File format: Portable Document Format (.pdf)
- Title of data: Data management software comparison table
- Description: Comparison of features between SODAR and related data management software.

Additional File 2

- File format: Portable Document Format (.pdf)

- Title of data: SODAR sample sheet editor
- Description: Figure consisting of screenshots of the SODAR sample sheet editor with its major features annotated.

## 6. Abbreviations

| | |
|---|---|
| API: | Application Programmable Interface |
| BAM: | Binary Alignment Map |
| BIDS: | Brain Imaging Data Structure |
| CDISC: | Clinical Data Interchange Standards Consortium |
| CUBI: | Core Unit Bioinformatics |
| DMF: | Data Management Framework |
| DRS: | Data Repository System |
| EGA: | European Genome-phenome Archive |
| ELN: | Electronic Laboratory Notebook |
| GEO: | Gene Expression Omnibus |
| GHGA: | German Human Genome-phenome Archive |
| GUI: | Graphical User Interface |
| HDF5: | Hierarchical Data Format v5 |
| HPO: | Human Phenotype Ontology |
| HTTP: | Hypertext Transfer Protocol |
| IDS: | Instrument-specific Data System |
| IGV: | Integrative Genomics Viewer |
| IRODS: | Integrated Rules-Oriented Data System |
| ISA: | Investigation Study Assay |
| JSON: | JavaScript Object Notation |
| LDAP: | Lightweight Directory Access Protocol |
| LIMS: | Laboratory Information Management System |
| MIT: | Massachusetts Institute of Technology (also commonly used with "MIT license") |
| NCBI: | National Center for Biotechnology Information |
| NFDI: | Nationale Forschungsdateninfrastruktur (German National Research Data Infrastructure) |
| OBO: | Open Biological and Biomedical Ontologies |
| OMIM: | Online Mendelian Inheritance in Man |
| OpenBIS: | Open Biology Information System |
| OWL: | Web Ontology Language |
| PAM: | Pluggable Authentication Mechanism |
| PEP: | Portable Encapsulated Projects |
| RBAC: | Role-Based Access Control |
| RDM: | Resource Data Management |
| REST: | Representational State Transfer |
| SDM: | Scientific Data Management |

SDMS: Scientific Data Management System
SODAR: System for Omics Access and Retrieval
TSV: Tabular Separated Values
UCSC: University of California Santa Cruz
VCF: Variant Call Format
WebDAV: Web-based Distributed Authoring and Versioning)
XML: Extensible Markup Language

## 7. Competing Interests

The authors declare that they have no competing interests.

### *7.1 Funding*

This work has been supported by the Ministry of Education and Research (BMBF), as part of the National Research Initiative 'Mass Spectrometry in Systems Medicine' (MSCoreSys), grant-ID 031L0220A (MSTARS) and by the Deutsche Forschungsgemeinschaft (DFG, German Research Foundation) – project-ID 427826188 – SFB 1444.

### *7.2 Author's Contributions*

Conceptualization: MN, MH, DB. Funding Acquisition: DB. Methodology: MH, MN. Project Administration: MH, DB. Resources: DB. Software: MN, MH, OS, PP. Supervision: MH, DB. Writing and Editing: all authors

## 8. Acknowledgements

The authors thank all internal users, particularly CUBI members, for their feedback. Some icons from OpenMoji.org were used in the figures.

## 9. References

[1] Gonzales A, Peres-Neto PR. Data curation: Act to staunch loss of research data. Nature 520(7548):436. 2015; doi:10.1038/520436c.

[2] Wilkinson MD, Dumontier M, Aalbersberg IJ, et al. The FAIR Guiding Principles for scientific data management and stewardship. Sci. Data 3:160018. 2016; doi:10.1038/sdata.2016.18.

[3] Donner E. Research data management systems and the organization of universities and research institutes: A systematic literature review. Journal of Librarianship and Information Science. 2022; doi:10.1177/09610006211070282.

[4] Machina HK, Wild DJ. Electronic Laboratory Notebooks Progress and Challenges in Implementation. Journal of Laboratory Automation. 2013;18(4):264-268; doi:10.1177/2211068213484471.

[5] Wolstencroft K, Owen S, Krebs O, et al. SEEK: a systems biology data and model management platform. BMC Syst Biol 9, 33. 2015; doi:10.1186/s12918-015-0174-y.

[6] King G. An Introduction to the Dataverse Network as an Infrastructure for Data Sharing. Sociological Methods & Research, 36(2). 2007; doi:10.1177/0049124107306660.

[7] Smeele T, Westerhof L. Using iRODS to manage, share and publish research data: Yoda. Proc. iRODS 2018 User Group Meeting, Durham NC, University of North Carolina. 2018.

[8] Tryka KA, Hao L, Sturcke A, et al. NCBI's Database of Genotypes and Phenotypes: dbGaP. Nucleic Acids Research, Volume 42, Issue D1. 2014; doi:10.1093/nar/gkt1211.

[9] Haug K, Cochrane K, Nainala VC, et al. MetaboLights: a resource evolving in response to the needs of its scientific community. Nucleic Acids Research, Volume 48, Issue D1. 2020; doi:10.1093/nar/gkz1019.

[10] Clough E, Barrett T. The Gene Expression Omnibus Database. Statistical Genomics. Methods in Molecular Biology, vol 1418. 2016; doi:10.1007/978-1-4939-3578-9_5.

[11] Van der Velde KJ, Imhann F, Charbon B, et al. MOLGENIS research: advanced bioinformatics data software for non-bioinformaticians. Bioinformatics, Volume 35, Issue 6. 2019; doi:10.1093/bioinformatics/bty742.

[12] Acevedo F, Arriaga V, Bass V, et al. Zendro Documentation. https://zendro-dev.github.io/. Accessed 7 Mar 2023.

[13] Sansone S-A, Rocca-Serra P, Field D, et al. Toward interoperable bioscience data. *Nat Genet.* 2012; doi:10.1038/ng.1054.

[14] Sheffield NC, Stolarczyk M, Reuter VP, Rendeiro AF. Linking big biomedical datasets to modular analysis with Portable Encapsulated Projects. *GigaScience*. 2021; doi:10.1093/gigascience/giab077.

[15] Gorgolewski KJ, Auer T, Calhoun VD, et al. The brain imaging data structure, a format for organizing and describing outputs of neuroimaging experiments. Sci. Data 3:160044. 2016.; doi:10.1038/sdata.2016.44.

[16] Facile R, Muhlbradt EE, Gong M, et al. Use of Clinical Data Interchange Standards Consortium (CDISC) Standards for Real-world Data: Expert Perspectives From a Qualitative Delphi Survey. JMIR Med Inform 10(1):e30363. 2022; doi:10.2196/30363.

[17] The HDF Group. Hierarchical Data Format, version 5. 1997-2023. https://www.hdfgroup.org/HDF5/. Accessed 21 Mar 2023.

[18] Bischof J, Wilke A, Gerlach W, et al. Shock: Active Storage for Multicloud Streaming Data Analysis. Proc. 2015 IEEE/ACM 2nd International Symposium on Big Data Computing (BDC), Limassol, Cyprus. 2015; doi:10.1109/BDC.2015.40.

[19] Ernst M, Fuhrmann P, Gasthuber M, et al. dCache, a distributed storage data caching system. Proc. CHEP 2001: international conference on computing in high energy and nuclear physics, Beijing (China). 2001.

[20] Hedges M, Blanke T, Hasan A. Rule-based curation and preservation of data: A data grid approach using iRODS. *Future Generation Computer Systems*. 2009; doi:10.1016/j.future.2008.10.003.

[21] Chiang G-T, Clapham P, Qi G, Sale K, Coates G. Implementing a genomic data management system using iRODS in the Wellcome Trust Sanger Institute. *BMC Bioinformatics*. 2011; doi:10.1186/1471-2105-12-361.

[22] Courtot M, Gupta D, Liyanage I, et al. BioSamples database: FAIRer samples metadata to accelerate research data management. *Nucleic Acids Research*. 2022; doi:.

[23] Mohr C, Friedrich A, Wojnar D, et al. qPortal: A platform for data-driven biomedical research. PLoS ONE 13(1): e0191603. 2018; doi:10.1371/journal.pone.0191603.

[24] Barillari C, Ottoz DSM, Fuentes-Serna JM, et al. openBIS ELN-LIMS: an open-source database for academic laboratories, Bioinformatics, Volume 32, Issue 4. 2016; doi:10.1093/bioinformatics/btv606.

[25] Carpi N, Minges A, Piel M. eLabFTW: An open source laboratory notebook for research labs. JOSS. 2017; doi:10.21105/joss.00146.

[26] Kuhn RM, Haussler D, Kent JW. The UCSC genome browser and associated tools. Briefings in Bioinformatics, Volume 14, Issue 2. 2013; doi:10.1093/bib/bbs038.

[27] Obermayer B, Holtgrewe M, Nieminen M, et al. SCelVis: exploratory single cell data analysis on the desktop and in the cloud. PeerJ 8:e8607; doi:10.7717/peerj.8607.

[28] Robinson JT, Thorvaldsdóttir H, Winckler W, et al. Integrative genomics viewer. Nat Biotechnol 29, 24–26. 2011; doi:10.1038/nbt.1754.

[29] The German Human Genome-Phenome Archive. https://www.ghga.de/. Accessed 28 Mar 2023.

[30] Freeberg MA, Fromont LA, D'Altri T, et al. The European Genome-phenome Archive in 2021. Nucleic Acids Research, Volume 50, Issue D1. 2022; doi:10.1093/nar/gkab1059.

[31] Nieminen M, Stolpe O, Schumann F, et al. SODAR Core: a Django-based framework for scientific data management and analysis web apps. *JOSS*. 2020; doi:10.21105/joss.01584.

[32] Kuhring M, Nieminen M, Kirwan J, et al. AltamAltamISA: a Python API for ISA-Tab files. *JOSS*. 2019; doi:10.21105/joss.01610.

[33] Ferraiolo DF, Kuhn DR, Chandramouli R. Role-Based Access Control, Second Edition. Artech House, 2006; ISBN-13:978-1-59693-113-8.

[34] Smeele T, Smeele C. Davrods, an Apache WebDAV interface to iRODS. *Proc. iRODS 2016 User Group Meeting*. 2016.

[35] Köhler S, Gargano M, Matentzoglu N, et al. The Human Phenotype Ontology in 2021. Nucleic Acids Research, Volume 49, Issue D1. 2021; doi:10.1093/nar/gkaa1043.

[36] Hamosh A, Scott AF, Amberger J, et al. Online Mendelian Inheritance in Man (OMIM), a knowledgebase of human genes and genetic disorders. Nucleic Acids Research, Volume 33, Issue suppl_1. 2005; doi:10.1093/nar/gki033.

[37] Federhen S. The NCBI Taxonomy database. Nucleic Acids Research, Volume 40, Issue D1. 2012; doi:10.1093/nar/gkr1178.

[38] Holtgrewe M, Stolpe O, Nieminen M, et al. VarFish: comprehensive DNA variant analysis for diagnostics and research. *Nucleic Acids Research*. 2020; doi:10.1093/nar/gkaa241.

[39] Stolpe O, Nieminen M, Obermayer B, et al. Kiosc: an integrated platform for managing bioinformatics data analysis containers. *Submitted*.

Click here to access/download;Table;Additional File 1.pdf

| (yes)=can be implemented within framework | | SDMS | | | | ELN | DRS | | DMF | |
|---|---|---|---|---|---|---|---|---|---|---|
| | | SODAR | qPortal | FAIRDom Seek | OpenBIS ELN-LIMS | eLabFTW | Dataverse | Yoda | Molgenis | Zendro |
| **SDMS features** | | | | | | | | | | |
| 1. Overarching | | | | | | | | | | |
| 1.a | Structure into projects/folders | yes | yes | yes | yes | yes | yes | yes | yes | yes |
| 1.b | Access control | yes | yes | yes | yes | yes | yes | yes | yes | yes |
| 1.c | Automation possible via API | yes | yes | yes | yes | yes | yes | yes | yes | yes |
| 1.d | Open standards / formats | yes | no | yes | no | yes | yes | yes | yes | yes |
| 2. Planning | | | | | | | | | | |
| 2.a | Structured recording of assays/experiments | yes | yes | yes | yes | limited | no | no | (yes) | (yes) |
| 2.b | Flexible definition of studies/experiments | yes | limited | yes | limited | yes | no | no | (yes) | (yes) |
| 2.c | Controlled vocabulary | yes | yes | yes | yes | no | no | no | (yes) | (yes) |
| 2.d | Ontologies | yes | no | yes | no | no | no | no | (yes) | (yes) |
| 3. Data collection | | | | | | | | | | |
| 3.a | Storage of files possible | yes | yes | yes | yes | yes | yes | yes | (yes) | no |
| 3.b | Many / large files | yes | no | no | limited | no | yes | yes | (yes) | no |
| 4. Data analysis | | | | | | | | | | |
| 4.a | Meta data API | yes | no | yes | yes | yes | yes | yes | yes | yes |
| 4.b | Mass data files API | yes | no | yes | limited | no | yes | yes | no | no |
| **5. Further features** | | | | | | | | | | |
| **5.a ELN** | | | | | | | | | | |
| 5.a.i | Flexible data entry text/table/pictures | no | no | no | yes | yes | no | no | no | no |
| **5.b DRS** | | | | | | | | | | |
| 5.b.i | Host public data repositories | no | no | yes | no | no | yes | yes | (yes) | (yes) |
| **5.c DMF** | | | | | | | | | | |
| 5.c.i | Easy creation of tables | no | no | no | no | no | no | no | yes | yes |
| 5.c.ii | User-centric data entry masks | limited | yes | limited | limited | limited | no | no | yes | yes |
| 5.c.iii | Predefined components, e.g., for data analysis | no | no | no | yes | no | no | no | yes | no |

Figure 1 Click here to access/download;Figure;Figure_1.pdf

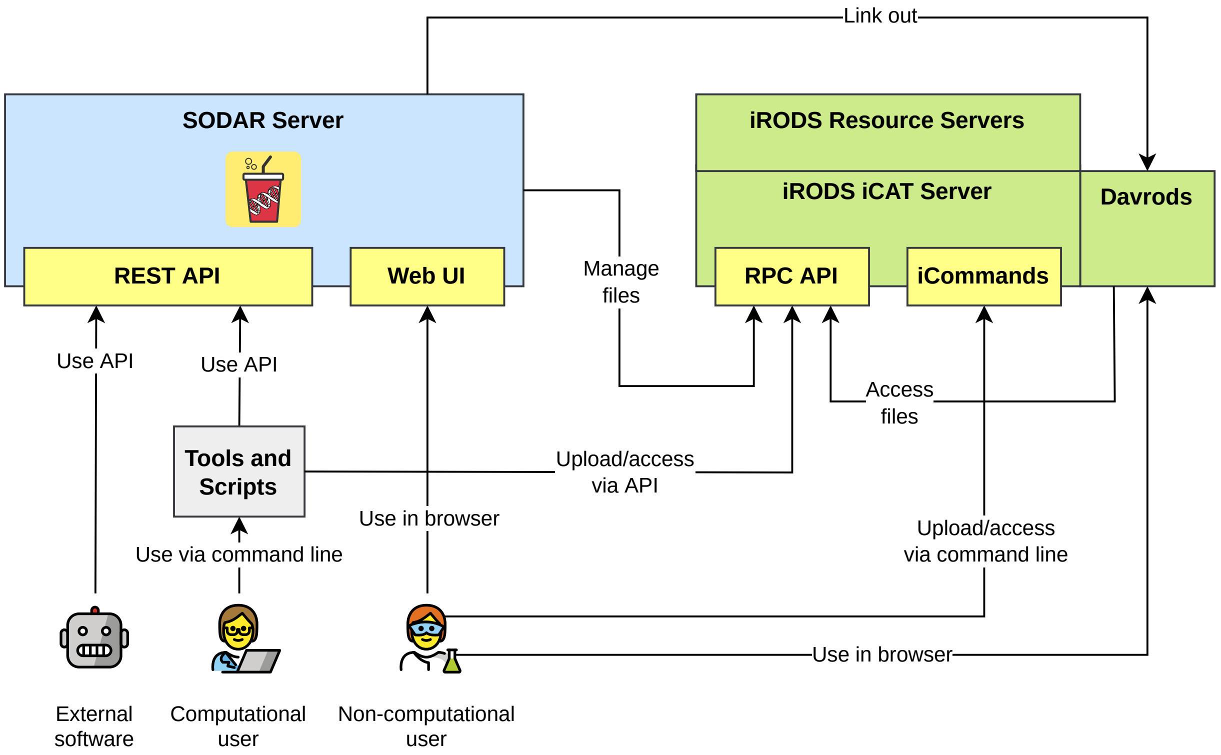

Figure 2

Click here to access/download;Figure;Figure_2.pdf

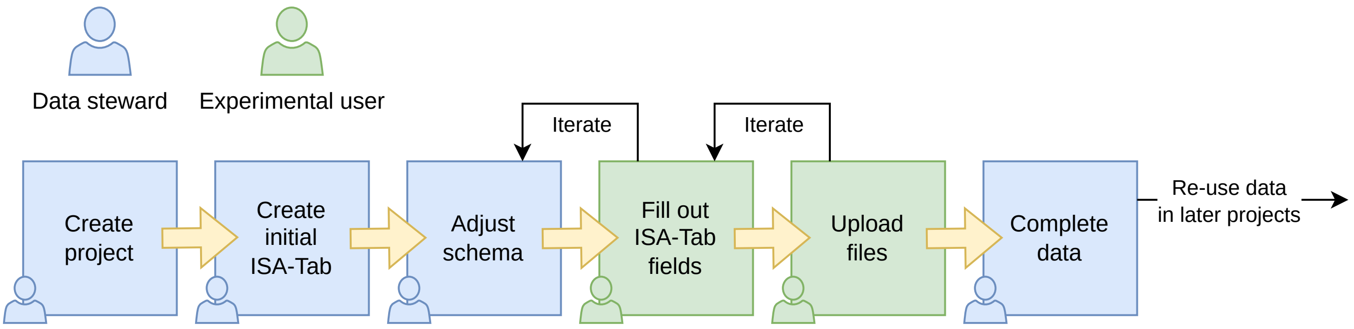

Click here to access/download;Figure;Figure_3.pdf

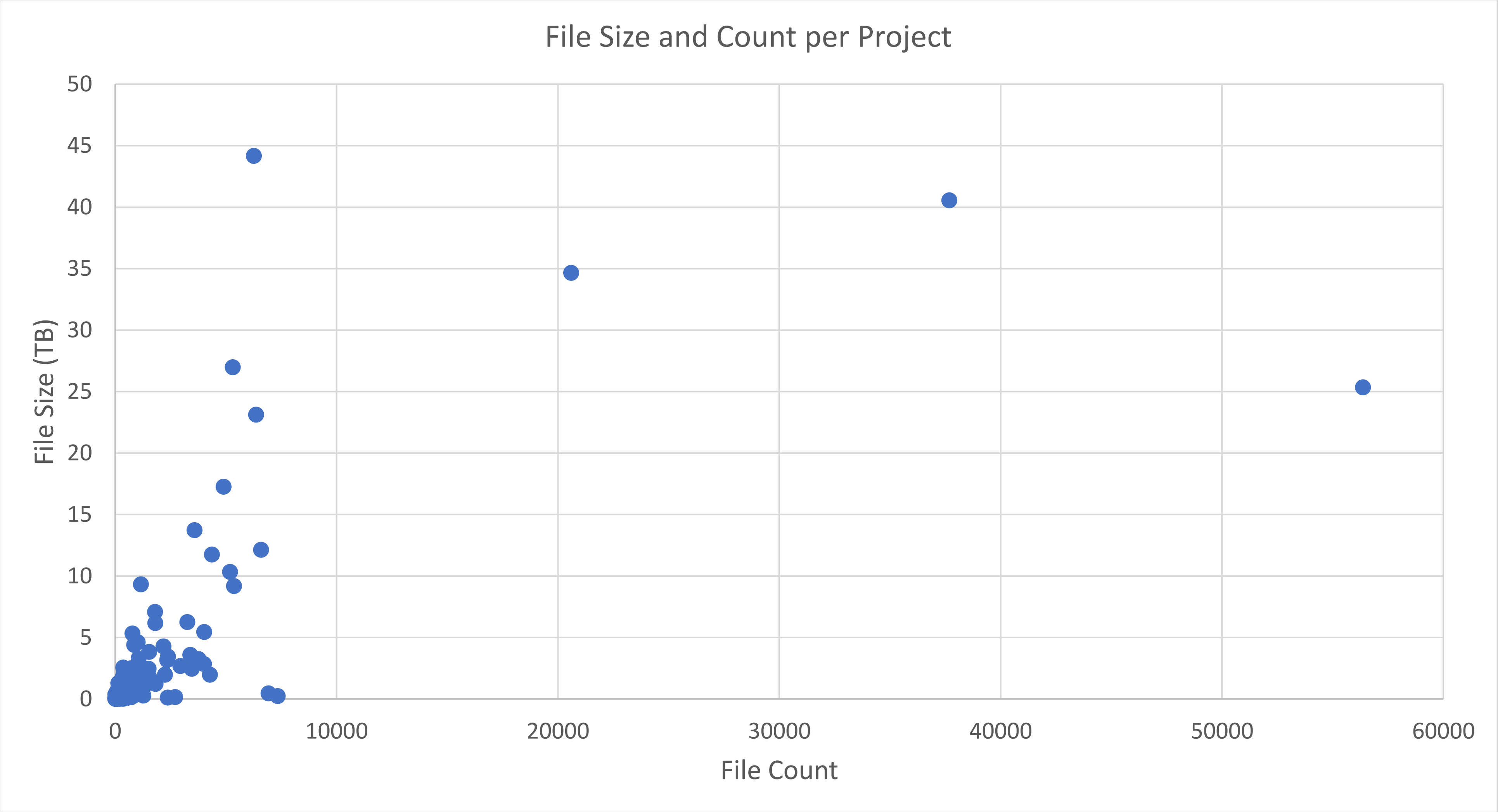

Additional File 2

Click here to access/download;Figure;Additional File 2.pdf

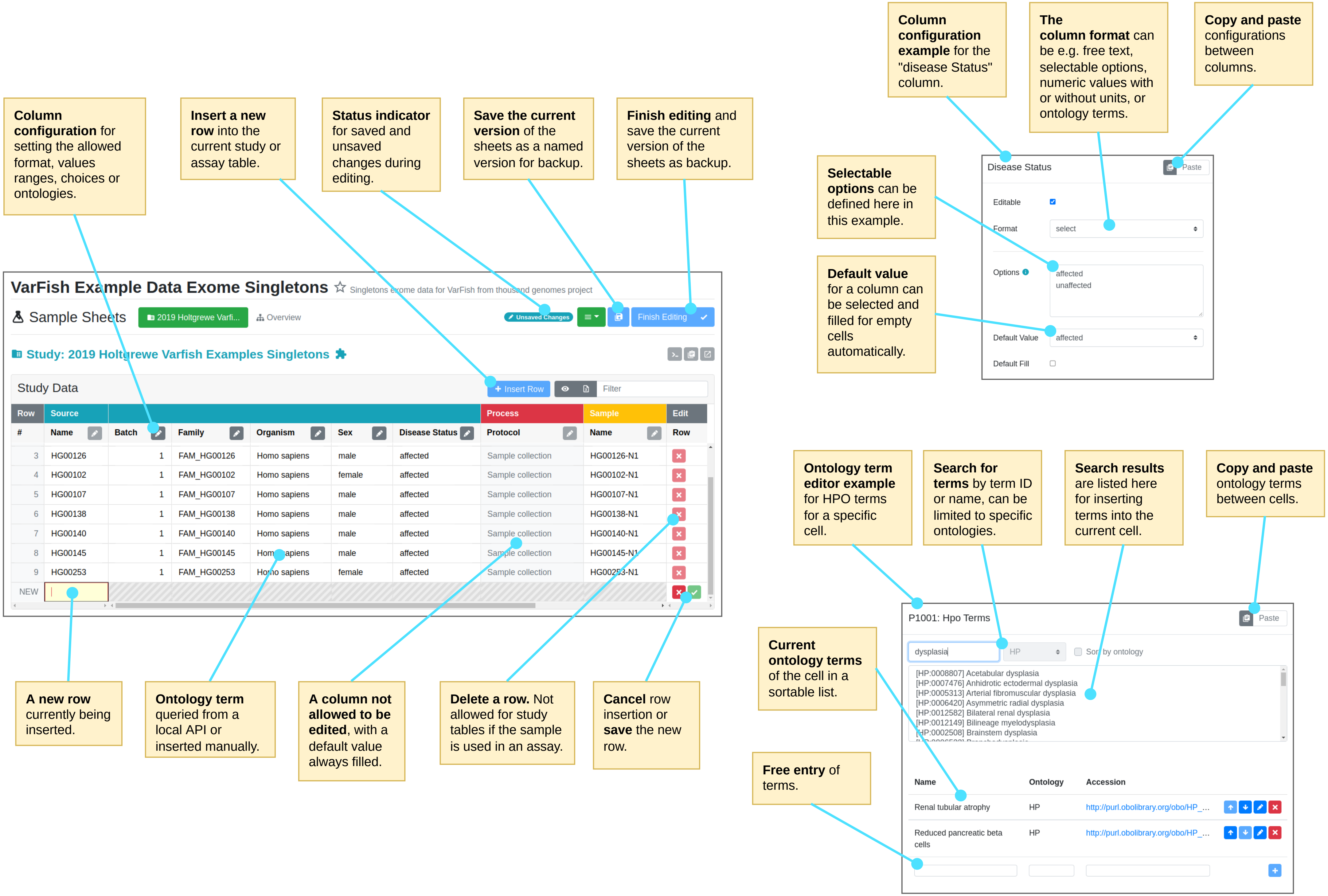

Supplement: giad052_GIGA-D-22-00194_Revision_1 [file giad052_giga-d-22-00194_revision_1.pdf]
