# Supplementary material for: SODAR: managing multiomics study data and metadata
Source: Gigascience. 2023 Jul 27;12:giad052. doi: 10.1093/gigascience/giad052 (PMC10373112; doi:10.1093/gigascience/giad052)
Supplement: giad052_Supplemental_Files [file giad052_supplemental_files.zip › Additional File 1.pdf]

(yes)=can be implemented within framework		SDMS				ELN	DRS		DMF	
		SODAR	qPortal	FAIRDom Seek	OpenBIS ELN-LIMS	eLabFTW	Dataverse	Yoda	Molgenis	Zendro
**SDMS features**										
1. Overarching										
1.a	Structure into projects/folders	yes	yes	yes	yes	yes	yes	yes	yes	yes
1.b	Access control	yes	yes	yes	yes	yes	yes	yes	yes	yes
1.c	Automation possible via API	yes	yes	yes	yes	yes	yes	yes	yes	yes
1.d	Open standards / formats	yes	no	yes	no	yes	yes	yes	yes	yes
2. Planning										
2.a	Structured recording of assays/experiments	yes	yes	yes	yes	limited	no	no	(yes)	(yes)
2.b	Flexible definition of studies/experiments	yes	limited	yes	limited	yes	no	no	(yes)	(yes)
2.c	Controlled vocabulary	yes	yes	yes	yes	no	no	no	(yes)	(yes)
2.d	Ontologies	yes	no	yes	no	no	no	no	(yes)	(yes)
3. Data collection										
3.a	Storage of files possible	yes	yes	yes	yes	yes	yes	yes	(yes)	no
3.b	Many / large files	yes	no	no	limited	no	yes	yes	(yes)	no
4. Data analysis										
4.a	Meta data API	yes	no	yes	yes	yes	yes	yes	yes	yes
4.b	Mass data files API	yes	no	yes	limited	no	yes	yes	no	no
**5. Further features**										
**5.a ELN**										
5.a.i	Flexible data entry text/table/pictures	no	no	no	yes	yes	no	no	no	no
**5.b DRS**										
5.b.i	Host public data repositories	no	no	yes	no	no	yes	yes	(yes)	(yes)
**5.c DMF**										
5.c.i	Easy creation of tables	no	no	no	no	no	no	no	yes	yes
5.c.ii	User-centric data entry masks	limited	yes	limited	limited	limited	no	no	yes	yes
5.c.iii	Predefined components, e.g., for data analysis	no	no	no	yes	no	no	no	yes	no
