# Supplementary material for: SODAR: managing multiomics study data and metadata
Source: Gigascience. 2023 Jul 27;12:giad052. doi: 10.1093/gigascience/giad052 (PMC10373112; doi:10.1093/gigascience/giad052)
Supplement: giad052_Supplemental_Files [file giad052_supplemental_files.zip › Additional File 2.pdf]

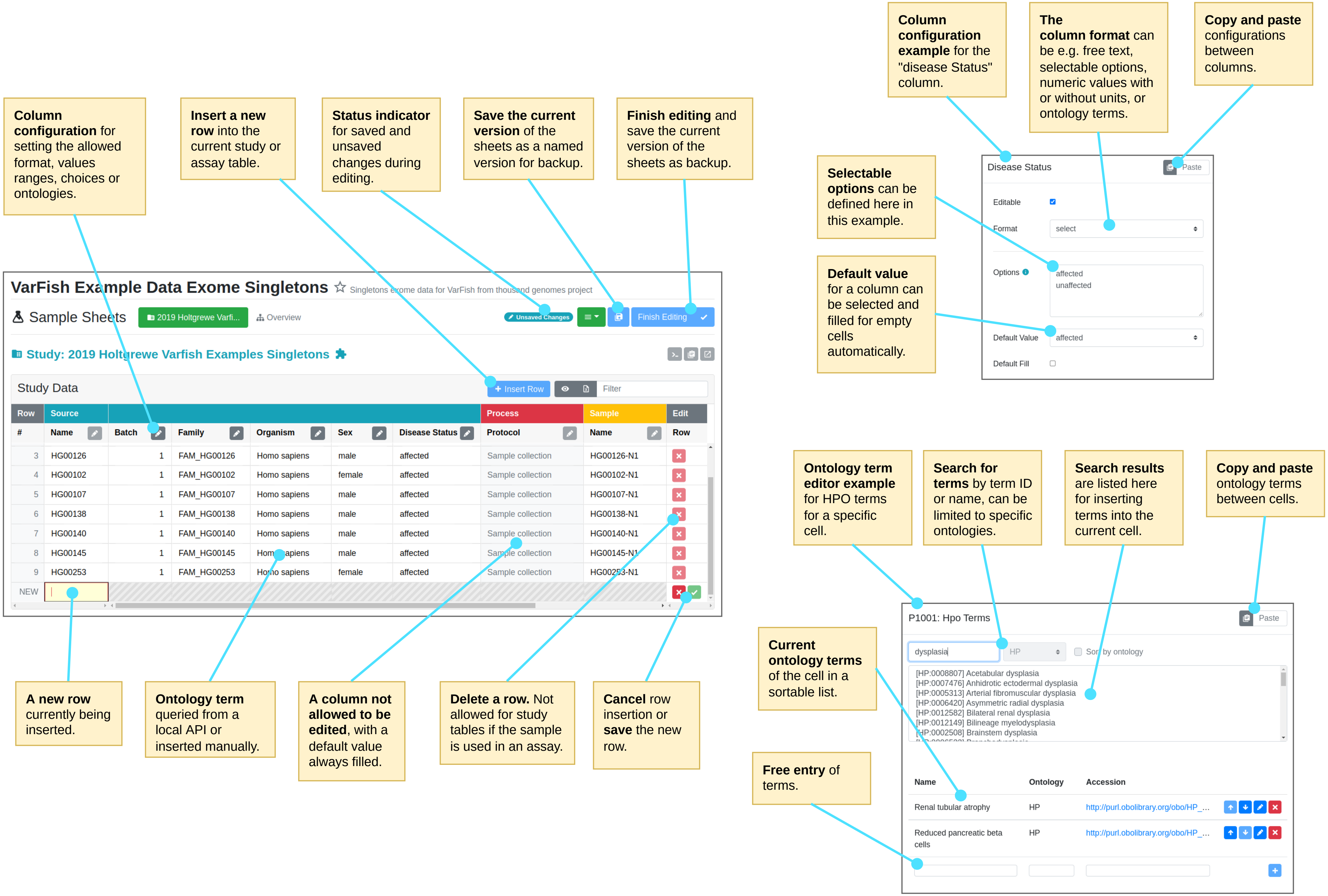

Column configuration example for the "disease Status" column.
The column format can be e.g. free text, selectable options, numeric values with or without units, or ontology terms.
Copy and paste configurations between columns.
Column configuration for setting the allowed format, values ranges, choices or ontologies.
Insert a new row into the current study or assay table.
Status indicator for saved and unsaved changes during editing.
Save the current version of the sheets as a named version for backup.
Finish editing and save the current version of the sheets as backup.
Selectable options can be defined here in this example.
Default value for a column can be selected and filled for empty cells automatically.
Disease Status
Paste
Editable
Format
select
Options
affected
unaffected
Default Value
affected
Default Fill
VarFish Example Data Exome Singletons
Singletons exome data for VarFish from thousand genomes project
Sample Sheets
2019 Holtgrewe Varfi...
Overview
Unsaved Changes
Finish Editing
Study: 2019 Holtgrewe Varfish Examples Singletons
Study Data
Insert Row
Filter
Row
Source
Process
Sample
Edit
#
Name
Batch
Family
Organism
Sex
Disease Status
Protocol
Name
Row
3 HG00126 1 FAM_HG00126 Homo sapiens male affected Sample collection HG00126-N1
4 HG00102 1 FAM_HG00102 Homo sapiens female affected Sample collection HG00102-N1
5 HG00107 1 FAM_HG00107 Homo sapiens male affected Sample collection HG00107-N1
6 HG00138 1 FAM_HG00138 Homo sapiens male affected Sample collection HG00138-N1
7 HG00140 1 FAM_HG00140 Homo sapiens male affected Sample collection HG00140-N1
8 HG00145 1 FAM_HG00145 Homo sapiens male affected Sample collection HG00145-N1
9 HG00253 1 FAM_HG00253 Homo sapiens female affected Sample collection HG00253-N1
NEW
Ontology term editor example for HPO terms for a specific cell.
Search for terms by term ID or name, can be limited to specific ontologies.
Search results are listed here for inserting terms into the current cell.
Copy and paste ontology terms between cells.
Current ontology terms of the cell in a sortable list.
Free entry of terms.
P1001: Hpo Terms
Paste
dysplasia
HP
Sort by ontology
[HP:0008807] Acetabular dysplasia
[HP:0007476] Anhidrotic ectodermal dysplasia
[HP:0005313] Arterial fibromuscular dysplasia
[HP:0006420] Asymmetric radial dysplasia
[HP:0012582] Bilateral renal dysplasia
[HP:0012149] Bilineage myelodysplasia
[HP:0002508] Brainstem dysplasia
Name
Ontology
Accession
Renal tubular atrophy
HP
http://purl.obolibrary.org/obo/HP_...
Reduced pancreatic beta cells
HP
http://purl.obolibrary.org/obo/HP_...
A new row currently being inserted.
Ontology term queried from a local API or inserted manually.
A column not allowed to be edited, with a default value always filled.
Delete a row. Not allowed for study tables if the sample is used in an assay.
Cancel row insertion or save the new row.
