# Supplementary material for: SODAR: managing multiomics study data and metadata
Source: Gigascience. 2023 Jul 27;12:giad052. doi: 10.1093/gigascience/giad052 (PMC10373112; doi:10.1093/gigascience/giad052)
Supplement: giad052_Response_to_Reviewer_Comments_Original_Submission [file giad052_response_to_reviewer_comments_original_submission.pdf]

Author's Response To Reviewer Comments

Close

Dear Hongling Zhou,

Thank you very for the reviews and for giving us the chance to revise our manuscript. We have carefully considered the comments and answered the questions of both reviewers and improved the manuscript, accordingly, see details below in our answers to the individual reviewers' comments, questions and suggestions. You can find the original response by you and the editors in black below and our answers in red.

We also thank you for extending the deadline for resubmission. It took us longer to appropriately respond to the reviewers' issues than we hoped for. We have done comprehensive updates to the manuscript and improved the SODAR software for addressing the reviewers' concerns. As a highlight, the manuscript now contains a greatly extended section on data management software categories and comparison. The SODAR software has been also updated to address the reviewers' concerns (a total addition of six thousand lines of code).

We have also greatly extended the projects in the SODAR demo server.

Editor:

The paper may need serious proofreading by an English native speaker, reviewers think it rushed and verbose.

Answer:

We have carefully reviewed the resubmitted manuscript for language and reading flow. We ask the reviewers to consider the updated text. If the reviewers think that issues persist, then we will address them subsequently.

Reviewer #1:

The authors developed the SODAR tool, which supports multi-omics integration studies. This is a great tool that has a user-friendly interface and supports multi-omics integration. However, I have several concerns that need to be addressed before this manuscript can be considered to be published.

Answer:

Thank you for your time and input in writing your review. We think that we have addressed your points in this letter and the manuscript.

Reviewer #1:

How does the SODAR handle the multi-omics data that are from different samples? For example, the gut microbiome data from stool samples and proteomics data from blood samples, which may be from the

same person but collected at different dates.

Answer:

This point is already addressed by the ISA-Tab file format and more a question of data model than of the SODAR system. The example given by the reviewer could be modeled by having the “person” as the ISA source and the different stool samples as ISA sample stemming from the same ISA source. The individual ISA samples can be annotated with the date each. Each ISA sample can then be subjected to an individual ISA assay, e.g., one “metagenome” and one “metatranscriptome” assay.

Reviewer #1:

Since SODAR supports cell editing, so how does it make the metadata and expression data consistent automatically?

Answer:

The user can update the meta data and create “invalid data” here, e.g., destroy links between the meta data and mass data files. We have updated the SODAR user interface software such that it will notify the user about such broken links such that corrective action can be taken.

Some information about linking meta and mass data can be found in the manual:

- https://sodar-server.readthedocs.io/en/latest/metadata_advanced.html

The sample sheet editor user interface is already keeping the structure of the meta data itself consistent. For example, the editor does not allow removing a source or sample from the “study” table if it is referenced by a sample in an “assay table”. Also, the ISA data model requires us to keep rows redundant with the same information in certain cases. For example, if there is a “split” in processes at some point, the rows left of it relating to the same “split source” material must be equal. The SODAR sample sheet editor ensures such invariants. This way, intrinsic consistency is kept automatically.

A full list of changes to the source code can be found here in the changelog.

- https://sodar-server.readthedocs.io/en/latest/sodar_changelog.html
- https://sodar-core.readthedocs.io/en/latest/changelog.html

Reviewer #1:

The authors claim that the SODAR can support multi-omics integration studies. However, I didn't find out how SODAR can do that. Could the authors give more descriptions about that?

Answer:

We have toned down the integration aspect as a response to the second reviewer.

Nevertheless, SODAR supports storing data from multi-omics studies through the ISA data model. For one study, users can store multiple assays, e.g., DNA-seq, RNA-seq, and metaproteomics. Results from each assay can be linked to the same original in the same study.

E.g., there can be the “Person 1” source, the “Person 1 blood” sample as well as “Person 1 stool”. The “Person 1 blood” can then be subjected to DNA and RNA sequencing in individual assays generating “Person 1 blood DNA FASTA” and “Person 1 blood RNA FASTA” files with subsequent analyses (variant

calling, RNA expression analysis) while a metaproteomics assay could be performed the “Person 1 stool” sample to yield “Person 1 stool metaproteomics” data.

Some more examples can be found here in the SODAR Server Documentation

- https://sodar-server.readthedocs.io/en/latest/metadata_recording.html?highlight=example#metadata-recording

The meta data of this study can be stored as ISA-Tab in SODAR and the mass data and individual result files stored in SODAR’s iRODS server. Users can then retrieve the sample sheet and secondary/tertiaray analysis files from SODAR and integrate them to, hypothetically, correlate genotype with RNA expression and stool metaproteomics results in a Jupyter notebook or R session.

Reviewer #2:

The reviewer thanks the authors for their efforts in producing the submitted manuscript.
The authors describe a django based web application designed to support data management.
The tool is built to support experimental metadata capture using the ISA format in its tsv form.
The tool relies on irods to manage data files associated with the experimental metadata.
The tool offers programmatic access via an API and clear front end.

Answer:

Thank you very much for your valuable time and your helpful comments and suggestions. We have improved our manuscript accordingly and addressed issues raised by the other reviewer. Please find details concerning the individual points you raised below.

Reviewer #2:

The title: "SODAR: enabling, modeling, and managing multi-omics integration studies" could be clearer. Being more concise "SODAR: standard compliant management of multi-omics studies " would deliver a better message.

Answer:

We have toned down the integration aspect and instead changed the title to the simpler “SODAR: managing multi-omics study-data and metadata” to address your concerns.

Reviewer #2:

Page 1 , Abstract: it would benefit from further refinement as there are several repetitions.

Answer:

We have updated the abstract.

Reviewer #2:

Check 3rd sentence for English. "ranging from....to..." , s/whereas/to/

"Scientists from diverse backgrounds also have different demands for interfacing with the data, ranging from computational users that need programmatic or command line access whereas non-computational users need graphical interfaces."

to:

"Scientists, with different backgrounds, ranging from computational scientists to wet-lab scientists, have different needs when it comes to data access, with programmatic interfaces being favoured by the former and graphical ones by the latter".

Instead of saying "under a permissive licence", be more explicit and plainly state "under MIT licence."

Answer:

We have updated this text.

Reviewer #2:

what is the difference between " data analysis and integration of data"?

Answer:

We have reworked this part of the manuscript.

Reviewer #2:

Repetition/redundancy in "An example of such complex study is (Esterhuyse et al., 2015) in infection biology, which will be used as an example below."

Answer:

We have removed this running example and particular part of the text.

Reviewer #2:

Use of term "modeling": using "plan" or "planning" may be better to remove any ambiguity about the nature of the modeling (statistical modeling, data modeling). Alternating, perfer 'representation' or 'representing'.
(the term model is repeated many times in the following sentences)

Answer:

Thank you for the thoughtful suggestions. We have adjusted and streamlined the terminology. We hope that it is now better/clearer.

Reviewer #2:

The statement "The most comprehensive standard for describing study metadata is the ISA-Tab format ..." is probably too strong. There are more formal (UML) models such as FUGE-OM

(https://doi.org/10.1038/nbt1347) or CDISC SDM & SDTM.

A more understated assessment such as "a popular standard, owing to its simplicity, is the ISA-Tab format"

"Alternatives include..." possibly cite other options for managing such complex datasets as seen with BIDS in neuroscience (Gorgolewski, K., Auer, T., Calhoun, V. et al. The brain imaging data structure, a format for organizing and describing outputs of neuroimaging experiments. Sci Data 3, 160044 (2016). https://doi.org/10.1038/sdata.2016.44) or why not mention HDF5 specification.

Answer:

We have restructured and rewritten these parts of the manuscript to address your concerns. In particular, we made the wording more "open" in that we do not aim a comprehensive description of available standards but simply mention some relevant ones in our context.

Reviewer #2:

This section could be improved by refining the transitions between the different ideas presented or organising the flow.

For example, by layout out the challenges of 1/ dealing with experimental metadata and 2/ dealing with digital objects produced by instruments, which have the characteristics outlined by the authors (volume, depth). Then review the technical solutions and then present the choices made by this implementation and possibly identify the selection criteria which led to choosing one specification over another.

Answer:

Thank you for raising this valid and important issue. We have thought long and hard how to appropriately address this. First, we restructured the text according to your suggestion in the layout.

Second, we have built on the categories by Machina & Wild (2013) to create a possible categorization of available software for management of scientific data. We think that this reflects the different aims and strengths of different software better than the original text and stresses how different aims and purposes shape different strengths in certain areas. We also think that this now explains how different software packages complement each other but also that there are no "hard lines" between features as some are shared between packages from different categories. Some packages attempt to provide comprehensive feature sets while others specialize in certain areas.

Reviewer #2:

Page 4: " Non-computational users can interface with SODAR using the graphical UI, whereas computational users can use command line interfaces and REST APIs from scripts and other external software."

Repeat from the abstract. I would suggest rephrasing to 'humanise' 'computational users' vs 'non-computation users', and identifying the function and roles in actual labs (bioinformaticians, data analysts, aka dry lab scientists) vs (experimentalists, wet-lab biologists).

Answer:

It is our opinion that the figure and wording therein is fine. We define dry lab scientist vs wet lab scientist as follows. Dry lab scientists are able to use the command line while wet lab scientists are "limited" to using graphical user interfaces. This distinction is not perfect, but we consider it useful for our purposes.

Reviewer #2:

Figure 1: same comment (in fact confirming by the choice of characters).

a question about the diagram: Is it the case that the Web UI does not talk to server via the API as done in some modern development. Probably highlight there the reliance on the Django framework.

Answer:

In the case of SODAR, the Web UI and the server are bundled together in a single Django project. The use of Django has been highlighted in the revised manuscript.

Reviewer #2:

Section 2.1
The first sentence needs attention, check the English. "for both serving for modeling experiments..."

Answer:

We have reviewed the manuscript text for this kind of language issues using support form native speakers.

Reviewer #2:

Also, there are systems (EBI Metabolights tools on their github repo, DataVerse, FAIRdom SEEK, Zendro...).

So the story telling should probably first talk about the survey of the existing and then only bring to arguments justifying new development.

Answer:

As mentioned above, we have reworked the text and think that the new version properly addresses the raised concerns and gives a proper and “fair” treatment of the considered software packages.

Reviewer #2:

Table 1.
It is odd to lump blanket statements for tools such as LIMS, ELN or 'Study Databases' without clearly stating which ones specifically have been evaluated.

It seems that one could formulate a table with very different results.

Question: How was selection bias controlled for?

Answer:

We have addressed this point together with the previous one. After giving the topic lots of consideration, we do not think that a bias free selection of tools is actually feasible. Finding open source tools through

Google or Github is hard. We have made an attempt to make a reasonable choice and think that we expressed this more clearly now in the manuscript.

We think that our solution in defining several "categories" of software packages based on the list of packages from Machina & Wild (2013) and considering important features inside each category gives a more objective treatment of the subject than our previous versions. We are interested in the reviewers' opinion about the new text and would welcome suggestions to improve it further.

Reviewer #2:

Page 5:
This section should be reorganised and each explanatory statement refined to add clarity. Case in point: "Arbitrary Experiments": Does experiment equate 'ISA.Assay'? is it akin to a Workflow or process Sequence ?

Answer:

Yes, the meaning of "experiment" in our manuscript directly maps to an "ISA.Assay". There, material/process flow can be expressed directly as directed acyclic graphics while back-references could be expressed informally by providing material/process names by "ISA Comments" or similar.

Reviewer #2:

Question: among the key feature that such a system should have to support the work of dry/wet lab scientists, surely, deposition to public repositories should be high on the list. Why is this absent?

Answer:

While there is no implementation of such a functionality from the WebUI, automatized export is of course possible using the API and in fact, in some projects, this is precisely how we do it. However, as SODAR does not focus on one data type, submission to public repositories is project specific. While some automatization is possible, in our experience publishing data requires often manual work.

We have added text to the manuscript clarifying this.

Reviewer #2:

Page 6:
typo: s/bioinfsormaticians/bioinformaticians/
punctuation: to be checked: missing commas make for a difficult read.
suggestion: simplify the role of 'experimentalists' in the context of SOBAR.
"They use the templates provided by the Data Stewards to instantiate a wet lab track and track its metadata."

Answer:

We have incorporated these language/wording issues.

Reviewer #2:

Question: How are data stewards trained in ISA-Tab?

Answer:

We have produced internal training material for data stewardship within our organization and projects. However, we consider this outside of the scope of this manuscript.

Reviewer #2:

Access to the demo tool gives the opportunity to use and test the component. While the UI is simple and intuitive, a number of limitations in the editing functionality make usage more difficult that it needs to be.

Answer:

The current set of supported features in the editor is based on tight collaboration with the 350+ users of our SODAR implementation. Limiting the number of features is a design choice to make the interface simpler, more intuitive and user-friendly, and we tried to pick these features which are commonly needed by our users. Of course, we will implement new features when the need arises.

Reviewer #2:

Page 7:
"of course, using the REST-API of SODAR, it is possible to automate these tasks"
Could the author produce a jupyter notebook showing how to do so?
It would be a nice addition and possibly a good resource that could facilitate uptake.

Answer:

We have added examples of API use into the SODAR Server manual here: https://sodar-server.readthedocs.io/en/dev/api_examples.html

These examples can also be used in a notebook or scripts to serve as basis for task automation in SODAR.

Reviewer #2:

Section 2-3:

page 8-9-10: this section could be streamlined and condensed to really focus on the interaction between shaping a sample processing & data acquisition workflow into a template which can be used by a wet lab scientists.
All this while allowing a markup with ontology terms.

Note: the ontology terms on the demo server do not resolve properly.

Answer:

Thank you for the remark, we have fixed this. The ontology term import to the demo server had failed earlier.

Reviewer #2:

Question: Why choosing Bioportal over other services, e.g. EBI OLS?

Answer:

There is no particular reason and we are/were not aware of any advantages or issue with either.

Reviewer #2:

Question: How can value-sets be constrained in SODAR?

Answer:

It is currently possible to constrain sets for simple string/numerical values, but not for ontology terms. A future upgrade to enable specifying allowed ontology term sets has been planned and can be found as issue #1615 in the sodar-server repository issue tracker.

Reviewer #2:

Question: ontology browser: it is unclear if the ontologies need to be loaded locally or if they are accessed via an API call to the relevant services ? Can the authors clarify this point?

Answer:

Ontology terms can be manually set, but for automated lookup in the UI, uploading ontologies into the system is required with system administrator capabilities.

Reviewer #2:

the demo server did not seem to allow it or I wasn't able. may be a figure showing the functionality would help?

Answer:

Local upload is done using the UI or management commands, but this functionality is limited to administrators.

Reviewer #2:

Page 11: Internal Usage Statistics
Question: it seems that the mean size of an experiment stored in SODAR is ~60 samples and about 10 files per sample.
These are relatively small sized studies.
Can the authors provide insights about the performance of the platform with large studies (several thousands of samples and above) ?

Answer:

We have added a figure showing number of files vs. Total file size for the projects. This illustrates that we have some large studies in our internal instance already (50+TB, 60+k files). The iRODS system can scale to millions of files overall, e.g., the Sanger instance stores thousands of Illumina sequencing runs with tens of thousands of files each with several PB of data.

Further, we have created a large sample project in the demo site with generated “mock” data that shows that the meta data system can handle thousands of samples.

Reviewer #2:

Question: Installation and deployment of SODAR.
Why the authors omit to mention that SODAR can be deployed via Docker? It seems useful information.

Answer:

This is mentioned in chapter 3.5, “SODAR Administration”.

Reviewer #2:

Question: AltamISA
Checking the library, it seems that development has stalled. It is a concern ?

Answer:

The library is developed by us SODAR authors and we keep it updated as we find identify limitations and bugs. The test coverage is at 95% and so far, we have encountered relatively few bugs per year. We consider the library to fit our scope very well and consider it stable and maintain it in case of bugs.

Reviewer #2:

Have the authors tested swapping AltamISA with ISA-API ?

Answer:

When we started out with ISA support in 2018, we found several concerns with ISA-API (we even started to make some fixes at https://github.com/mkuhring/isa-api/tree/cubi-hotfixes). One important feature was “round-tripping”, I.e., loading a file, writing it out again and getting more or less the same content. We checked on 2022-09-13 and the current main branch does not have working CI. Furthermore, a self-written library is much easier to extend for features such as extended validation. ISA-Tab files can be exported from SODAR and used with ISA-API.

Reviewer #2:

Is it at all possible ? could it be made via an adaptor of some sort ?

Answer:

This is not currently planned. However, if the need arises, it is possible to develop support in the future or

integrate a pull request from a third party.

Reviewer #2:

Can Altam ISA convert to ISA-JSON or other public repository compatible format to provide a capability to assist users disseminate their results?

Answer:

We have not yet implemented ISA-JSON support for Altamisa as we have not found the need for it. So far, we have not found any adopters of ISA-JSON besides the ISA-JSON authors but would be interested to learn about them.

SODAR currently focuses on storing studies while they are running. In our use case within a clinic environment, many projects' data cannot be deposited in public databases, in particular given our background in the German regulatory system. When contributing to public databases, we have written custom scripts to prepare GEO submissions, for example. As SODAR offers REST APIs and allows to export ISA-Tab we have found this sufficient.

Reviewer #2:

figure 3 should not be a supplementary material but a proper content as it is useful as showcasing SODAR UI and customization.

Answer:

As we understand it, including such a large figure should be done as a supplement according to this journal's conventions. We believe it is best to leave this to the editor to decide.

Close
