# Supplementary material for: SODAR: managing multiomics study data and metadata
Source: Gigascience. 2023 Jul 27;12:giad052. doi: 10.1093/gigascience/giad052 (PMC10373112; doi:10.1093/gigascience/giad052)
Supplement: giad052_Response_to_Reviewer_Comments_Revision_1 [file giad052_response_to_reviewer_comments_revision_1.pdf]

Author's Response To Reviewer Comments

Close

Dear editor and reviewers,

We thank you again for the feedback and a chance to review our manuscript. Our answers to reviewer comments for our second submission are detailed below.

Editor:

page 4, 2nd paragraph, first sentence: typo -> s/approaching itusing/approaching it using/

Answer:

This has been fixed.

Editor:

page 7, 2nd paragraph, suggested edit:

change from: "For publication, raw and processed data and metadata are deposited in scientific catalogues, study
databases and registries. An example is the BioSamples database for metadata [22]."

"to:
For publication, metadata and raw or processed data are deposited in scientific catalogues, study databases and registries. Examples are the BioSamples database for metadata [22] and Short Read Archive for raw sequencing data [citation needed]."

Answer:

This has been changed as suggested.

Editor:

1. this sentence makes a disservice to the manuscript: "Our work is
representative of the work typically done by core units in clinics. Clinical settings often deal with humans as their primary sample source. This implies controlled access of data, or not being allowed to share confidential data. Thus, developing support for hosting data in a public repository is not our aim. Likewise, uploading data to other public repositories has not been a priority. "

Two reasons:
- the first one is opening the can of worms of data governance and oversight of patient related information. I would steer clear of that in this piece.
- the second one is because i would flip the argument around. "While deposition to public repositories was not necessarily the priority, the development of an (almost, see below) ISA compliant system provides

such a capability should the data owner need it"

Answer:

We changed the section according to the proposal, although with slightly different wording.

Editor:

2. in the result section, or in the documentation, a welcome addition would be example of templates for non-sequencing based assays. For instance, since the authors mentioned their need to support proteomics and mass-spectrometry users, it would make sense to highlight the templates available. In other words, it would help the target audience of the manuscript locate 'metadata profile definitions' (somewhat akin to ISA configurations) for specific assay types. If I have missed it from the manuscript or the github repo, please ignore.

Answer:

We have added a non-sequencing-based metabolomics study “MTBLS691: MaHPIC Experiment E03” into the sodar-paper repository, as well as on the SODAR demo site.

Editor:

3. "dialectic" ISA format:
Several examples are available from the GitHub repository generally follow the ISA-Tab specifications but also introduce a `local` field: "Library Name". While such value would make sense in the official ISA specification, it is currently not supported. This leads to the creation of a diverging format.
It would be sensible to keep the "Library Name" as an presentation label (for display in the UI) and substitute it to "Labeled Extract Name" when exporting outside the database to the tab format, in order to retain compatibility with other ISA parsers and the official specifications.
It could be added as an output option to the Altam-ISA parser in case deposition to public repositories is needed (e.g. EMBL-Metabolights). This would go some way in helping 'Interoperability' and would not be too onerous a change.
Worth of note, I was recently made aware that ENA repository would be accepting submission in ISA-Tab and ISA-JSON format, hence raising this point to the authors.
Suggestion: clarify this in the Methods section.

Answer:

Thank you for this remark. We had a previous discussion on the ISA-tab google group where the ISA-tab authors appeared to be open to adding such an extension to the official standard. We only now realized that we never followed up on this, though, and just used this extension in the data.

Although we have used this extension in our data in the past, the “Library Name” field is not actually required by or hard-coded into SODAR to be included in any ISA-Tab. We have updated our example projects to omit this extension. Furthermore, in the latest SODAR release v0.13.4, the templates used for ISA-Tab creation also do not include this extension. In the future, we will introduce an administrator command in SODAR to force compatibility on our existing sample sheets by renaming the occurrences of this non-standard field.

Close
