# Supplementary material for: SODAR: managing multiomics study data and metadata
Source: Gigascience. 2023 Jul 27;12:giad052. doi: 10.1093/gigascience/giad052 (PMC10373112; doi:10.1093/gigascience/giad052)
Supplement: giad052_Response_to_Reviewer_Comments_Revision_2 [file giad052_response_to_reviewer_comments_revision_2.pdf]

Author's Response To Reviewer Comments

Close

Dear Editors,

Thank you for your feedback. The manuscript has been updated according to your comments. An editable .docx version was also included in this submission.

We registered previously missing CUBI software with Scicrunch to receive the requested RRID:s, which have been added into the "Methods" section. Please note that at the time of submission, these registrations have been accepted, but are still in the "curated" state. This means it may take a while from them to appear in their final form.

Please let us know if any further modifications are needed.

Regards,
-Mikko Nieminen

Close
