# Supplementary material for: SODAR: managing multiomics study data and metadata
Source: Gigascience. 2023 Jul 27;12:giad052. doi: 10.1093/gigascience/giad052 (PMC10373112; doi:10.1093/gigascience/giad052)
Supplement: giad052_Reviewer_2_Report_Original_Submission — Philippe Rocca-Serra -- 9/2/2022 Reviewed [file giad052_reviewer_2_report_original_submission.pdf]

Reviewer Report

Title: SODAR: managing multi-omics study data and metadata

Version: Original Submission **Date:** 9/2/2022

Reviewer name: Philippe Rocca-Serra

Reviewer Comments to Author:

The reviewer thanks the authors for their efforts in producing the submitted manuscript.The authors describe a django based web application designed to support data management.The tool is built to support experimental metadata capture using the ISA format in its tsv form.The tool relies on irods to manage data files associated with the experimental metadata.The tool offers programmatic access via an API and clear front end.Main comments:The title: "SODAR: enabling, modeling, and managing multi-omics integration studies" could be clearer.Being more concise "SODAR: standard compliant management of multi-omics studies " would deliver a better message.Page 1 , Abstract: it would benefit from further refinement as there are several repetitions.Check 3rd sentence for English. "ranging from....to..." , s/whereas/to/"Scientists from diverse backgrounds also have different demands for interfacing with the data, rangingfrom computational users that need programmatic or command line access whereas non-computationalusers need graphical interfaces."to:"Scientists, with different backgrounds, ranging from computational scientists to wet-lab scientists, have different needs when it comes to data access, with programmatic interfaces being favoured by the former and graphical ones by the latter".Instead of saying "under a permissive licence", be more explicit and plainly state "under MIT licence."Page 2, Introduction:what is the difference between " data analysis and integration of data"?Repetition/redundancy in "An example of such complex study is (Esterhuyse et al., 2015) in infection biology,which will be used as an example below. "Suggestion:Use of term "modeling": using "plan" or "planning" may be better to remove any ambiguity about the nature of the modeling (statistical modeling, data modeling). Alternating, perfer 'representation' or 'representing'.(the term model is repeated many times in the following sentences)The statement "The most comprehensive standard for describing study metadata is the ISA-Tab format ..." is probably too strong. There are more formal (UML) models such as FUGE-OM (https://doi.org/10.1038/nbt1347) or CDISC SDM & SDTM.A more understated assessment such as "a popular standard, owing to its simplicity, is the ISA-Tab format""Alternatives include..." possibly cite other options for managing such complex datasets as seen with BIDS in neuroscience (Gorgolewski, K., Auer, T., Calhoun, V. et al. The brain imaging data structure, a format for organizing and describing outputs of neuroimaging experiments. Sci Data 3, 160044 (2016). https://doi.org/10.1038/sdata.2016.44) or why not mention HDF5 specification.This section could be improved by refining the transitions between the different ideas presented or organising the flow.For example, by layout out the challenges of 1/ dealing with experimental metadata and 2/ dealing with digital objects produced by instruments, which have the characteristics outlined by the authors (volume, depth). Then review the technical solutions and then present the choices made by this implementation and possibly identify the selection criteria which led to choosing one specification over another.Results:Page 4: " Non-computational users can interface with SODAR using the graphical

UI, whereas computational users canuse command line interfaces and REST APIs from scripts and other external software."Repeat from the abstract. I would suggest rephrasing to 'humanise' 'computational users' vs 'non-computation users', and identifying the function and roles in actual labs (bioinformaticians, data analysts, aka dry lab scientists) vs (experimentalists, wet-lab biologists).Figure 1: same comment (in fact confirming by the choice of characters).a question about the diagram: Is it the case that the Web UI does not talk to server via the API as done in some modern development. Probably highlight there the reliance on the Django framework.Section 2.1The first sentence needs attention, check the English. "for both serving for modeling experiments..."Also, there are systems (EBI Metabolights tools on their github repo, DataVerse, FAIRdom SEEK, Zendro...).So the story telling should probably first talk about the survey of the existing and then only bring to arguments justifying new development.Table 1.It is odd to lump blanket statements for tools such as LIMS, ELN or 'Study Databases' without clearly stating which ones specifically have been evaluated.It seems that one could formulate a table with very different results.Question: How was selection bias controlled for?Page 5:This section should be reorganised and each explanatory statement refined to add clarity. Case in point:"Arbitrary Experiments": Does experiment equate 'ISA.Assay'? is it akin to a Workflow or process Sequence ?Question: among the key feature that such a system should have to support the work of dry/wet lab scientists, surely, deposition to public repositories should be high on the list. Why is this absent?Page 6:typo: s/bioinfsormaticians/bioinformaticians/punctuation: to be checked: missing commas make for a difficult read.suggestion: simplify the role of 'experimentalists' in the context of SOBAR."They use the templates provided by the Data Stewards to instantiate a wet lab track and track its metadata."Question: How are data stewards trained in ISA-Tab?Access to the demo tool gives the opportunity to use and test the component. While the UI is simple and intuitive, a number of limitations in the editing functionality make usage more difficult that it needs to be.Page 7:"of course, using the REST-API of SODAR, it is possible to automate these tasks"Could the author produce a jupyter notebook showing how to do so?It would be a nice addition and possibly a good resource that could facilitate uptake.Section 2-3:page 8-9-10: this section could be streamlined and condensed to really focus on the interaction between shaping a sample processing & data acquisition workflow into a template which can be used by a wet lab scientists.All this while allowing a markup with ontology terms.Note: the ontology terms on the demo server do not resolve properly.Question: Why choosing Bioportal over other services, e.g. EBI OLS?Question: How can value-sets be constrained in SODAR?Question: ontology browser: it is unclear if the ontologies need to be loaded locally or if they are accessed via an API call to the relevant services ? Can the authors clarify this point?the demo server did not seem to allow it or I wasn't able. may be a figure showing the functionality would help?Page 11: Internal Usage StatisticsQuestion: it seems that the mean size of an experiment stored in SODAR is ~60 samples and about 10 files per sample.These are relatively small sized studies.Can the authors provide insights about the performance of the platform with large studies (several thousands of samples and above) ?Methods:-------Question: Installation and deployment of SODAR.Why the authors omit to mention that SODAR can be deployed via Docker? It seems useful information.Question: AltamISAChecking the library, it seems that development has stalled. It is a concern ?Have the authors tested swapping AltamISA with ISA-API ?Is it at all possible ? could it be made via an adaptor of some sort ?Can Altam ISA convert to ISA-JSON or other public repository compatible format to provide a capability to assist users

disseminate their results?Comment:figure 3 should not be a supplementary material but a proper content as it is useful as showcasing SODAR UI and customization.

Level of Interest

Please indicate how interesting you found the manuscript: Choose an item.

Quality of Written English

Please indicate the quality of language in the manuscript: Choose an item.

Declaration of Competing Interests

Please complete a declaration of competing interests, considering the following questions:

- Have you in the past five years received reimbursements, fees, funding, or salary from an organisation that may in any way gain or lose financially from the publication of this manuscript, either now or in the future?
- Do you hold any stocks or shares in an organisation that may in any way gain or lose financially from the publication of this manuscript, either now or in the future?
- Do you hold or are you currently applying for any patents relating to the content of the manuscript?
- Have you received reimbursements, fees, funding, or salary from an organization that holds or has applied for patents relating to the content of the manuscript?
- Do you have any other financial competing interests?
- Do you have any non-financial competing interests in relation to this paper?

If you can answer no to all of the above, write 'I declare that I have no competing interests' below. If your reply is yes to any, please give details below.

I declare that I have no competing interests

I agree to the open peer review policy of the journal. I understand that my name will be included on my report to the authors and, if the manuscript is accepted for publication, my named report including any attachments I upload will be posted on the website along with the authors' responses. I agree for my report to be made available under an Open Access Creative Commons CC-BY license (http://creativecommons.org/licenses/by/4.0/). I understand that any comments which I do not wish to be included in my named report can be included as confidential comments to the editors, which will not be published.

Choose an item.

To further support our reviewers, we have joined with Publons, where you can gain additional credit to further highlight your hard work (see: https://publons.com/journal/530/gigascience). On publication of this paper, your review will be automatically added to Publons, you can then choose whether or not to claim your Publons credit. I understand this statement.

Yes Choose an item.
