# Supplementary material for: SODAR: managing multiomics study data and metadata
Source: Gigascience. 2023 Jul 27;12:giad052. doi: 10.1093/gigascience/giad052 (PMC10373112; doi:10.1093/gigascience/giad052)
Supplement: giad052_Reviewer_2_Report_Revision_1 — Philippe Rocca-Serra -- 4/20/2023 Reviewed [file giad052_reviewer_2_report_revision_1.pdf]

Reviewer Report

Title: SODAR: managing multi-omics study data and metadata

Version: Revision 1 **Date:** 4/20/2023

Reviewer name: Philippe Rocca-Serra

Reviewer Comments to Author:

The reviewer thank the authors for their efforts and extensive rework of the manuscripts, and for delivering this software stack.minor corrections:------------------page 4, 2nd paragraph, first sentence: typo -> s/approaching itusing/approaching it using/page 7, 2nd paragraph, suggested edit:change from: "For publication, raw and processed data and metadata are deposited in scientific catalogues, studydatabases and registries. An example is the BioSamples database for metadata [22].""to:For publication, metadata and raw or processed data are deposited in scientific catalogues, studydatabases and registries. Examples are the BioSamples database for metadata [22] and Short Read Archive for raw sequencing data [citation needed].""important clarifications:------------------------1. this sentence makes a disservice to the manuscript: "Our work isrepresentative of the work typically done by core units in clinics. Clinical settings often deal with humansas their primary sample source. This implies controlled access of data, or not being allowed to shareconfidential data. Thus, developing support for hosting data in a public repository is not our aim.Likewise, uploading data to other public repositories has not been a priority. "Two reasons:- the first one is opening the can of worms of data governance and oversight of patient related information. I would steer clear of that in this piece.- the second one is because i would flip the argument around. "While deposition to public repositories was not necessarily the priority, the development of an (almost, see below) ISA compliant system provides such a capability should the data owner need it"2. in the result section, or in the documentation, a welcome addition would be example of templates for non-sequencing based assays. For instance, since the authors mentioned their need to support proteomics and mass-spectrometry users, it would make sense to highlight the templates available. In other words, it would help the target audience of the manuscript locate 'metadata profile definitions' (somewhat akin to ISA configurations) for specific assay types. If I have missed it from the manuscript or the github repo, please ignore.3. "dialectic" ISA format:Several examples are available from the GitHub repository generally follow the ISA-Tab specifications but also introduce a `local` field: "Library Name". While such value would make sense in the official ISA specification, it is currently not supported. This leads to the creation of a diverging format.It would be sensible to keep the "Library Name" as an presentation label (for display in the UI) and substitute it to "Labeled Extract Name" when exporting outside the database to the tab format, in order to retain compatibility with other ISA parsers and the official specifications.It could be added as an output option to the Altam-ISA parser in case deposition to public repositories is needed (e.g. EMBL-Metabolights). This would go some way in helping 'Interoperability' and would not be too onerous a change.Worth of note, I was recently made aware that ENA repository would be accepting submission in ISA-Tab and ISA-JSON format, hence raising this point to the authors.Suggestion: clarify this in the Methods section.Also, it seems the following example is missing 'Assay Name' and 'Raw Data File' fields:https://raw.githubusercontent.com/bihealth/sodar-

paper/main/GSE96583_PBMC_Single-Cell_Demo_Project/a_PBMC_test_scRNAseq_nucleotide_sequencing.txt

Level of Interest

Please indicate how interesting you found the manuscript: Choose an item.

Quality of Written English

Please indicate the quality of language in the manuscript: Choose an item.

Declaration of Competing Interests

Please complete a declaration of competing interests, considering the following questions:

- Have you in the past five years received reimbursements, fees, funding, or salary from an organisation that may in any way gain or lose financially from the publication of this manuscript, either now or in the future?
- Do you hold any stocks or shares in an organisation that may in any way gain or lose financially from the publication of this manuscript, either now or in the future?
- Do you hold or are you currently applying for any patents relating to the content of the manuscript?
- Have you received reimbursements, fees, funding, or salary from an organization that holds or has applied for patents relating to the content of the manuscript?
- Do you have any other financial competing interests?
- Do you have any non-financial competing interests in relation to this paper?

If you can answer no to all of the above, write 'I declare that I have no competing interests' below. If your reply is yes to any, please give details below.

I am the maintainer of the ISA specifications and supporting python API, ISA-API.

I agree to the open peer review policy of the journal. I understand that my name will be included on my report to the authors and, if the manuscript is accepted for publication, my named report including any attachments I upload will be posted on the website along with the authors' responses. I agree for my report to be made available under an Open Access Creative Commons CC-BY license (http://creativecommons.org/licenses/by/4.0/). I understand that any comments which I do not wish to be included in my named report can be included as confidential comments to the editors, which will not be published.

Choose an item.

To further support our reviewers, we have joined with Publons, where you can gain additional credit to further highlight your hard work (see: https://publons.com/journal/530/gigascience). On publication of this paper, your review will be automatically added to Publons, you can then choose whether or not to claim your Publons credit. I understand this statement.

Yes Choose an item.
